# Phosphoglycerate dehydrogenase promotes pancreatic cancer development by interacting with eIF4A1 and eIF4E

**DOI:** 10.1186/s13046-019-1053-y

**Published:** 2019-02-11

**Authors:** Xuhui Ma, Boya Li, Jie Liu, Yan Fu, Yongzhang Luo

**Affiliations:** 1 The National Engineering Laboratory for Anti-Tumor Protein Therapeutics, Tsinghua University, Beijing, 100084 China; 2 Beijing Key Laboratory for Protein Therapeutics, Tsinghua University, Beijing, 100084 China; 3 Cancer Biology Laboratory, School of Life Sciences, Tsinghua University, Beijing, 100084 China

**Keywords:** PHGDH, eIF4A1, eIF4E, Translation initiation, Pancreatic cancer development

## Abstract

**Background:**

Pancreatic cancer is one of the most malignant cancers. The overall 5-year survival rate of its patients is 8%, the lowest among major cancer types. It is very urgent to study the development mechanisms of this cancer and provide potential targets for therapeutics design. Glucose, one of the most essential nutrients, is highly exploited for aerobic glycolysis in tumor cells to provide building blocks. However, the glucose consumption manner in pancreatic cancer cells is unclear. And the mechanism of the substantial metabolic pathway promoting pancreatic cancer development is also unrevealed.

**Methods:**

^13^C_6_ glucose was used to trace the glucose carbon flux and detected by mass spectrum. The expressions of PHGDH were determined in cells and pancreatic adenocarcinomas. Knockdown and overexpression were performed to investigate the roles of PHGDH on pancreatic cancer cell proliferation, colony formation and tumor growth. The mechanisms of PHGDH promoting pancreatic cancer development were studied by identifying the interacting proteins and detecting the regulatory functions on translation initiations.

**Results:**

Pancreatic cancer cells PANC-1 consumed large amounts of glucose in the serine and glycine de novo synthesis. Phosphoglycerate dehydrogenase (PHGDH) highly expressed and controlled this pathway. Knockdown of PHGDH significantly attenuated the tumor growth and prolonged the survival of tumor bearing mice. The pancreatic adenocarcinoma patients with low PHGDH expression had better overall survival. Mechanistically, knockdown of PHGDH inhibited cell proliferation and tumorigenesis through disrupting the cell-cell tight junctions and the related proteins expression. Besides catalyzing serine synthesis to activate AKT pathway, PHGDH was found to interact with the translation initiation factors eIF4A1 and eIF4E and facilitated the assembly of the complex eIF4F on 5’ mRNA structure to promote the relevant proteins expression.

**Conclusion:**

Besides catalyzing serine synthesis, PHGDH promotes pancreatic cancer development through enhancing the translation initiations by interacting with eIF4A1 and eIF4E. Inhibiting the interactions of PHGDH/eIF4A1 and PHGDH/eIF4E will provide potential targets for anti-tumor therapeutics development.

**Electronic supplementary material:**

The online version of this article (10.1186/s13046-019-1053-y) contains supplementary material, which is available to authorized users.

## Background

Pancreatic cancer is one of the most malignant cancers. Almost all patients who suffer from pancreatic cancer develop metastases and die [1]. The overall 5-year survival rate of pancreatic cancer patients is 8%, the lowest among major cancer types [2]. Pancreatic cancer is predominantly ductal adenocarcinoma and contains oncogenic Kras mutation, which plays a critical role in tumor initiation [3, 4]. The mutation of Kras exhibits a vital role in controlling tumor metabolism through stimulation of glucose uptake and promotion of ribose biogenesis [4]. In the present study, we observe that pancreatic cancer cells consume a large amount of glucose but produce far less molar of pyruvate compared with the theoretical value. However, the substantial mechanism, by which glucose-derived carbon is exploited, is not clearly understood. We propose that the unique pattern of glucose metabolism in pancreatic cancer cells is a sally port to understand this malignant cancer type.

In contrast to normal differentiated cells, tumor cells proliferate under conditions of nutrient stress, so they have to switch their metabolism preference from completely mitochondrial oxidative phosphorylation to aerobic glycolysis to meet the requirements of large amounts of energy and building blocks [5, 6]. Despite the universal alterations in glucose metabolism observed in tumors [5–7], diversion of glycolytic flux into amino acid synthesis pathways can be essential during tumor development [8, 9]. Serine and glycine are important amino acids synthesized in the branching pathway derived from glycolysis process. They are primarily essential elements for proteins synthesis, and moreover, can be converted to other biomolecules for cell proliferation, such as purine, thymidine, choline and methionine [10–12].

Phosphoglycerate dehydrogenase (PHGDH) is the first enzyme in the serine and glycine de novo biosynthetic pathway [13]. It catalyzes the transformation of glycolytic intermediate 3-phosphoglycerate into phosphohydroxypyruvate [14, 15], which is subsequently being converted to serine and being eventually transformed into glycine via serine hydroxymethyltransferase 1 (SHMT1) [10, 16]. Elevated protein expression of PHGDH are found in 21% of melanoma samples [8], 70% of estrogen receptor (ER)-negative [11] and triple-negative [8] breast cancers. High expression of PHGDH is also found to be associated with poor prognosis in breast cancer and lung adenocarcinomas [17, 18]. As a member of a complex regulating network, PHGDH is also under the tight controls. A member of PKC family, PKCζ, acts as a suppressor of PHGDH through phosphorylating the key residues of PHGDH to inhibit its enzymatic activity [19]. In non-small cell lung cancer (NSCLC) cells, a transcription factor NRF2 controls the expression of PHGDH and other key serine/glycine biosynthesis enzyme genes to support glutathione and nucleotide production [20]. In glioma, PHGDH interacts with and stabilizes the oncogenic transcription factor FOXM1 to promote the proliferation, invasion and tumorigenicity of glioma cells [21]. The inhibitors of PHGDH reduce the production of glucose-derived serine and suppress the growth of PHGDH-dependent breast cancers in culture and in orthotopic xenograft tumors and concomitantly reduce the nucleotide synthesis [22, 23]. In the past decade, PHGDH is identified to contain a di-nucleotide binding domain, serving for NAD+/NADH and RNA binding [24–26], which implies that, in addition to enzymatic activity, PHGDH probably has more novel functions to be discovered. However, the defined functions of PHGDH in pancreatic cancer are barely understood. In this study, we clarified that the metabolic preference of glycolysis process was highly diverted into the serine and glycine de novo synthesis flux. A systematic investigation proved that PHGDH expression was critical for pancreatic cancer development. Mechanistically, PHGDH interacted with the translation initiation factors eIF4A1 and eIF4E and facilitated the assembly of the translation initiation complex eIF4F to promote the development of pancreatic cancer.

## Methods

### Cell lines and cell cultures

PANC-1, AsPC-1, HMEC and other cell lines were obtained from the American Type Culture Collection and were maintained in DMEM supplemented with 10% FBS plus 1% antibiotics (100 U/ml penicillin and 100 mg/ml streptomycin, HyClone Thermo scientific).

### Antibodies and chemical reagents

Antibodies of PHGDH, SHMT1, eIF4A1, eIF4G1 and eIF4E were purchased from Abcam; antibodies of ZO-1 and E-cadherin were purchased from Cell Signaling Technology. ^13^C_6_ glucose was purchased from Sigma-Aldrich (St. Louis, MO). Inhibitor CBR5884 was purchased from TOCIRS; inhibitor 4EGI-1 was purchased from Selleck; cisplatin and paclitaxel were purchased from J&K.

### LC-MS/MS

Cells were cultured in basal media with FBS. Media were changed to fresh DMEM or MEM 12 h before cell collection. In the ^13^C_6_ labeled experiment, after cells adherence, media were changed to glucose free DMEM containing 11 mM ^13^C_6_ glucose and cells were continuously cultured for 48 h. For metabolite collection, media from biological triplicates (in 3.5 cm dishes at 80% confluence) were fully aspirated, and 1 ml methanol was added on ice for 20–25 min after twice washes with cold PBS. Then cells and the metabolite-containing supernatants were collected into conical tubes. Insoluble materials in lysates were centrifuged at 2000 *g* for 10 min, and the resulting supernatant was evaporated using a CentriVap Concentrator (LABCONCO). Samples were re-suspended using 100 μl HPLC grade 80% acetonitrile for mass spectrometry. 10 μl were injected and analyzed using 6460 Triple Quad LC/MS system (Agilent Technology) coupled to a 1290 UPLC system (Agilent Technology). Data analysis was performed in Cluster3.0 and TreeViewer.

### Immunohistochemical assay

Tumor tissue microarrays containing pancreatic ductal adenocarcinoma clinical samples (Biomax, US) were deparaffinized and treated with 3% hydrogen peroxide for 10 min. Antigen retrieval was performed in 10 mmol/l sodium citrate buffer by heating for 15 min in a microwave oven. Then tumor tissue slides were stained with primary antibodies (1:200–1:400 dilution) at 4 °C for overnight.

### Lentivirus production and infection

The lentivector expression plasmids, the packaging vector pR8.74, the envelope plasmid pVSVG and the transfer plasmid SGEP [27] containing the short hairpin RNA (shRNA) species targeting sequences for PHGDH mRNA (5’GCCGCAGAACTCACTTGTGGAA3’) or SHMT1 mRNA (5’ATCAGAAGTGTATGTTAGTCAA3’), were co-transfected into HEK293T cells using PEI reagent (Polysciences Inc.). For stable over-expression lentivirus production, plasmid pLentiCMV was used as transfer plasmid. The viral supernatant was collected 72 h after transfection and filtered with 0.45 mm filter. Lentiviruses were concentrated using Lenti-Concentin virus precipitation solution (ExCell Bio) according to the manufacturer’s instructions.

### Proliferation assay

Cells were cultured in 96-well plate for 24 or 48 h. Then the media were replaced with fresh DMEM and 5% (*v*/v) CCK8 up to a final volume of 100 μl. Incubated the cells for 2 h and detected the absorbance at 450 nm.

### Colony formation assays

1 × 10^3^ tumor cells were inoculated in 60-mm dishes and cultured in DMEM or MEM supplemented with 10% FBS. 1 mM serine or glycine (Sigma-Aldrich) was respectively added into the culture media. Colonies were cultured up to 14 days and then stained with crystal violet for analysis.

### Immunoprecipitation and co-immunoprecipitation assays

Cells were collected and centrifuged at 3000 rpm for 5 min. Washed the collected cells with cold PBS and lysed with lysis buffer (20 mM Tris, pH 7.4, containing 0.5% NP40, 150 mM sodium chloride, 0.5 mM phenylmethylsulfonyl fluoride, and one protease inhibitor mixture tablet (Roche)) at 4 °C for 20 min. Then collected the supernatant by centrifuging at 14,000 rpm for 10 min and incubated with the antibodies (1 μg/ml) and protein A-Sepharose (Roche) at 4 °C for overnight. The protein A-Sepharose beads were washed with the lysis buffer for three times at 4 °C. Precipitated protein was treated with 5 × SDS sample buffer at 100 °C for 5 min. The samples were then applied to 10% SDS-PAGE and detected by western blot.

### Pull down assays

2 μg recombinant PHGDH protein (Abcam) and 2 μg anti-PHGDH antibody (Abcam) were added into the interacting buffer (20 mM Tris, pH 7.4, 150 mM sodium chloride and protease inhibitor mixture) at 4 °C for 2 h. The recombinant proteins of eIF4A1 (Abbexa), eIF4E (Abcam), eIF4G1 (NOVUS) and their mixture were respectively added into the tubes to interact with PHGDH at 4 °C for 2 h. Then added 20 μl protein G-Sepharose (Roche) at 4 °C for overnight. The protein G-Sepharose beads were washed with the lysis buffer for three times at 4 °C. Precipitated protein was treated with 5 × SDS sample buffer at 100 °C for 5 min. The samples were then applied to 10% SDS-PAGE and detected by western blot.

### 7-methyl-GTP pull-down assays

Cell lysates were incubated with 20 μl 7-Methyl-GTP (m^7^-GTP)-Sepharose 4B (Jena Biosciences) at 4 °C for overnight. Beads were washed three times with lysis buffer and re-suspended in SDS sample buffer at 100 °C for 5 min. The samples were then applied to 10% SDS-PAGE and detected by western blot.

### Immunofluorescence assays

Cells were fixed by 4% paraformaldehyde for 20 min at room temperature and then permeabilized with PBS containing 0.2% Triton X-100. After twice washes with PBS, the cells were blocked with PBS containing 10% normal goat serum at 37 °C for 30 min. After blocking, the cells were stained with different primary antibodies at 4 °C for overnight. After twice washes with PBS, the cells were stained with FITC-, Alexa 555- or Alexa 647-conjugated secondary antibodies, and nuclei were stained by DAPI. Confocal fluorescence imaging was performed on Olympus Fluoview laser scanning confocal imaging system (Olympus, Melville, NY). The images were analyzed with Nikon image software NIS-Elements AR 3.0.

### Polysomes profiling assays

Cells were cultured in 15 cm dishes to reach 80~90% confluence. Prior to collection, incubated cells with cycloheximide at a final concentration of 100 μg/ml in growth media for 5 min at 37 °C and 5% CO_2_. The following experimental process was performed as Gandin et al. described in their paper [28]. Extracted the polysome associated mRNA using Trizol according to the manufacturer’s instructions for further qRT-PCR analysis.

### Animal studies

All animal studies were approved by the Institutional Animal Care and Use Committees of Tsinghua University (project number: 14-LYZ1). Different constructed pancreatic cancer cells (5 × 10^6^) were inoculated subcutaneously into the right flank of 6–8 weeks old male BALB/c nude mice (Vital River, Beijing, China). Each group contained at least 6 mice. Tumor volumes were measured every 3 or 4 days and were calculated by the formula: volume = 0.52ab^2^ (“a” indicates the long diameter and “b” is the short diameter). For orthotopic tumor incubation, different constructed pancreatic cancer cells (5 × 10^6^) were inoculated into the pancreas of 3–4 weeks old male BALB/c nude mice by surgery. During the surgery, 100 μl antibiotic and 100 μl meloxicam were injected subcutaneously. After 7–8 weeks, mice were sacrificed and the livers and lungs were resected. Each group contained at least 6 mice.

### Survival analysis of TCGA datasets

The publicly available cBioPortal for Cancer Genomics (http://www.cbioportal.org/) and TCGA datasets were utilized to analyze *PHGDH* and *SHMT1* genes expression and overall survival in 178 pancreatic adenocarcinoma patients. Kaplan-Meier survival curves were used to determine the survival rate as a function of time, and survival differences were analyzed by a log-rank Mantel-Cox test using GraphPad Prism.

### Statistical analysis

Experimental data were presented as mean ± standard deviation (SD). Statistical differences were assessed by a two-tailed Student’s t-test; *p* < 0.05 was considered to be significant.

## Results

### Glycolytic metabolism is highly diverted into the serine and glycine synthesis flux in PANC-1 cells

**Fig. 1:**
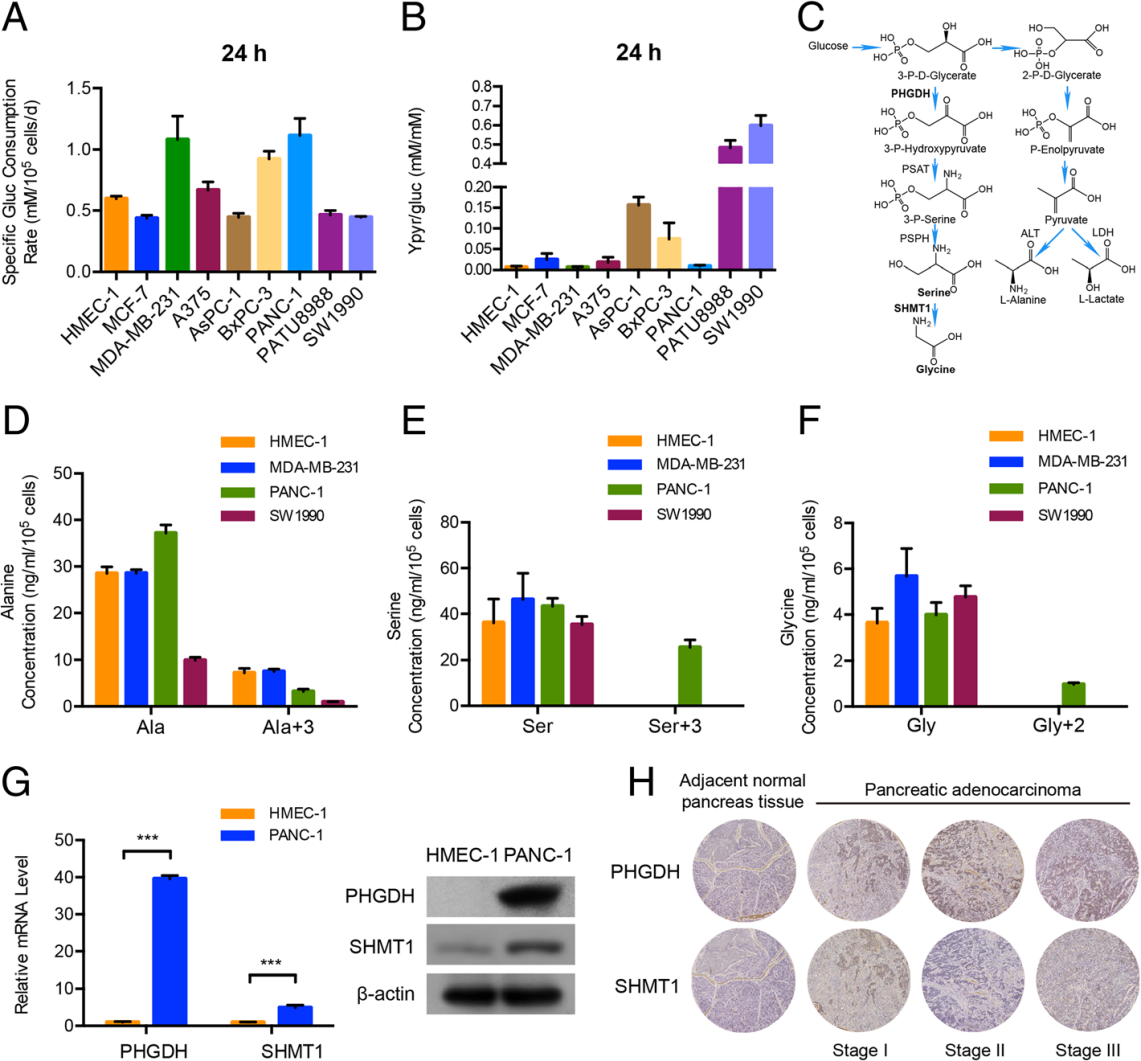
Glycolytic metabolism is highly diverted into the serine and glycine synthesis flux in PANC-1 cells. **a** and **b** Glucose consumption rates and pyruvate yield rates in different breast cancer, melanoma and pancreatic cancer cells in 24 h. **c** Schematic of glucose glycolytic metabolism divided into serine and glycine at 3-P-D-glycerate through PHGDH and SHMT1, and alanine at pyruvate through ALT respectively. **d**, **e** and**f** Carbon-unlabeled and labeled contents of alanine, serine and glycine in HMEC, MDA-MB-231, PANC-1 and SW1990 cells. **g** qRT-PCR and western blot analysis of the PHGDH and SHMT1 mRNA and protein levels in HMEC and PANC-1 cells. **h** Immunohistochemical analysis of PHGDH and SHMT1 expression levels in 24 pancreatic adenocarcinomas and 4 normal adjacent tissues. 4 representative cores with different malignancies are shown in the figure. Data are representative of at least three independent experiments. *p* value: Student’s t-test; ***p < 0.001. Columns: mean; bars: SD

Metabolic pathways alterations commonly occur in the process of a normal cell developing neoplasia. The consumption rate of the essential nutrient glucose is greatly different between neoplastic cells and normal cells. Firstly, we detected the glucose consumption rates in normal human endothelial cells, breast cancer cells, melanoma cells and pancreatic cancer cells. Among these cells, breast cancer cells MDA-MB-231, pancreatic cancer cells BxPC-3 and PANC-1 consumed much more glucose compared with the normal and other cancer cells (Fig. 1a). In the glucose catabolic process, pyruvate acts as a hinge product between glycolysis process and tricarboxylic acid (TCA) cycle. The content of pyruvate reflects the carbon flux from glucose. Thus we detected the concentration of pyruvate and calculated its yield rate to glucose in these cells. Results showed that, in MDA-MB-231 and PANC-1 cells, the relative transformation of glucose to pyruvate were the lowest (Fig. 1b). These results suggest that much more glucose in MDA-MB-231 and PANC-1 cells is diverted to produce glycolysis intermediates and further derivatives rather than pyruvate compared with other cancer cells. Based on glucose glycolytic pathways (Fig. 1c), we detected the products of branching pathway derived from glycolysis process, serine, glycine, alanine, and meanwhile, pyruvate, to find out which pathway occupied the largest carbon flux from glucose. ^13^C_6_ glucose in place of normal glucose was added into the culture media to trace the glucose carbon flux, so the newly synthesized serine had a mass-shift of 3 (M + 3) and glycine had a mass-shift of 2 (M + 2) due to the incorporation of glucose-derived ^13^C via 3-P-D-glycerate. Pyruvate and alanine had a mass-shift of 3 (M + 3). After 48 h, we detected the concentrations of all carbon labeled alanine (Ala+ 3), serine (Ser + 3), glycine (Gly + 2) and pyruvate (Pyr + 3) by means of targeted liquid chromatography mass spectrometry (LC-MS/MS) in the HMEC cells, MDA-MB-231 cells, PANC-1 cells and SW1990 pancreatic cancer cells. As the results showed, the Ala+ 3 was detected in all four cell lines (Fig. 1d), however, the Ser + 3 and Gly + 2 were only detected in the PANC-1 cells (Fig. 1e and f) and none Pyr + 3 was detected because of its low concentration (Additional file 1: Figure S1). Although the levels of unlabeled serine and glycine were obviously detected among the four cell lines, the solely accumulated Ser + 3 and Gly + 2 (Fig. 1e and f) demonstrated that the glucose in the glycolysis process was highly consumed in the serine and glycine synthesis pathway in PANC-1 cells. We considered this metabolic characteristic would be a sally port for pancreatic cancer study.

PHGDH is the first enzyme responsible for serine and glycine de novo synthesis [14, 15] and SHMT1 catalyzes the reversible inter-conversion of serine to glycine [10, 16]. Corresponding to the high production of serine and glycine, the mRNA and protein levels of PHGDH and SHMT1 in PANC-1 cells were much higher than that in the normal cells (Fig. 1g). Moreover, PHGDH was also found to highly express in patient samples and positively related with the tumor malignancy (Fig. 1h), suggesting this metabolic preference was exploited in clinical pancreatic adenocarcinomas.

### Knockdown of PHGDH attenuates pancreatic cancer development through inhibiting cell proliferation and tumorigenesis

**Fig. 2:**
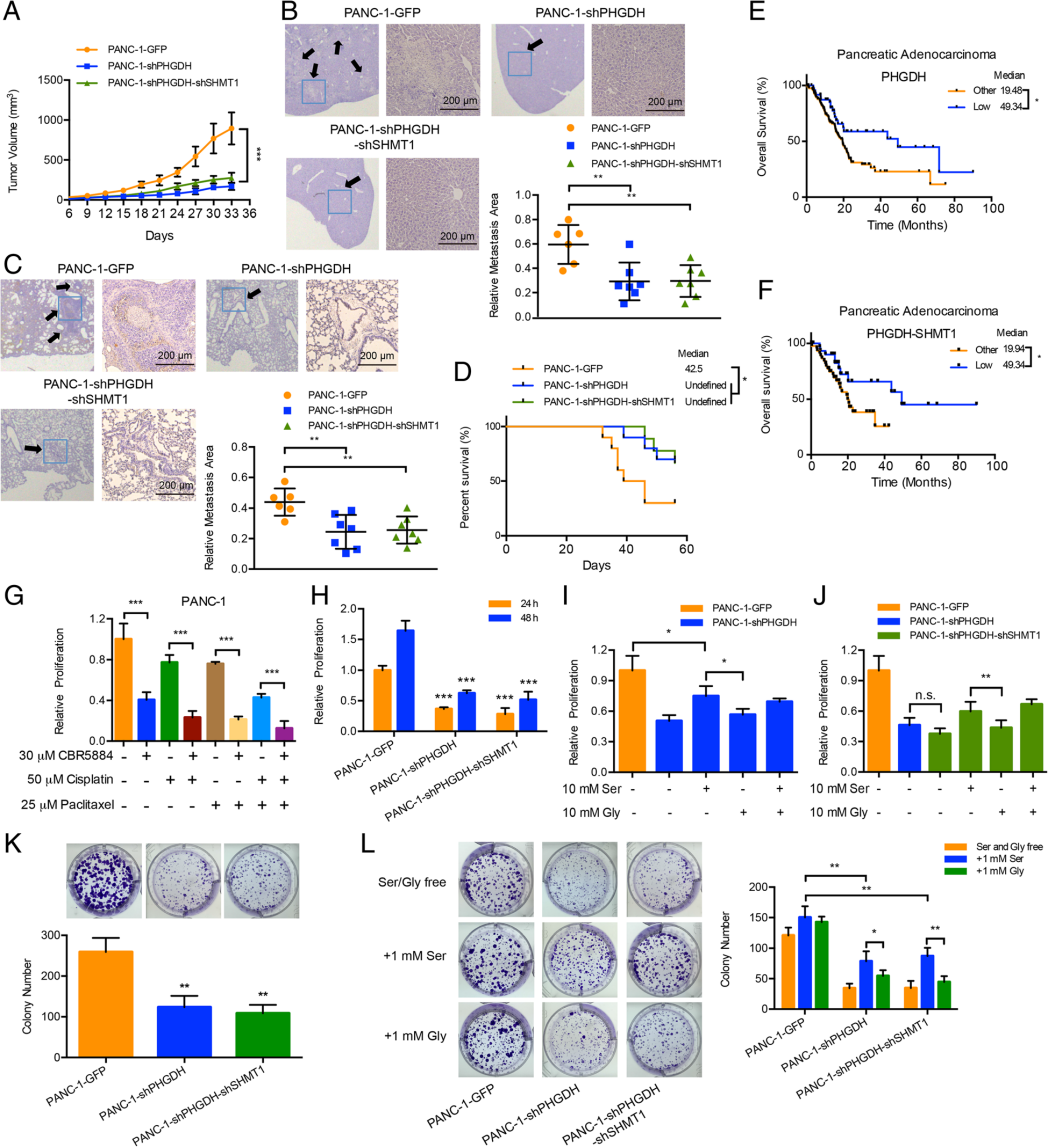
Knockdown of PHGDH attenuates pancreatic cancer development through inhibiting cell proliferation and tumorigenesis. **a** Nude mice were subcutaneously inoculated with the constructed PANC-1 cells. Tumor sizes were measured every 3 or 4 days. Each value is the mean ± SEM of determinations in at least 6 nude mice of each group. **b, c** and **d** Nude mice were orthotopically inoculated with the constructed PANC-1 cells. **b** and **c** Liver and lung metastases (arrows, metastatic foci) were detected with HE staining. Scale bars, 200 μm. The quantitative results represented the relative area of metastatic foci to the whole tissue slide; **d** The survival statuses were analyzed for 8 weeks. Each group at least contained 6 nude mice. **e** and **f** Kaplan-Meier curve for overall survival of the pancreatic adenocarcinoma patients based on (**e**) PHGDH mRNA and (**f**) PHGDH and SHMT1 mRNAs expression in the TCGA dataset. The blue line represented the patients with (**e**) low PHGDH and (**f**) low PHGDH and low SHMT1 expression levels (z-score < − 0.5); the black line represented other patients (z-score ≥ − 0.5). **e** *n* = 178. **f** *n* = 80. **g** Relative proliferation results of PANC-1 cells under the treatments of 30 μM PHGDH inhibitor CBR5884, 50 μM cisplatin and/or 25 μM paclitaxel for 24 h. **h** Relative proliferation results of the constructed PANC-1 cells for 24 and 48-h cultures. **i** and **j** Relative proliferation results of the constructed PANC-1 cells under the treatments of 10 mM serine and/or 10 mM glycine. **k** and **l** Colony formation results of the constructed PANC-1 cells under (**k**) the normal conditions and (**l**) the conditions of serine and glycine free (up), 1 mM serine supply (middle) and 1 mM glycine supply (down) respectively. Data are representative of at least three independent experiments. *p* value: Student’s t-test; *p < 0.05,**p < 0.01, ***p < 0.001. Columns: mean; bars: SD

To investigate the impacts of serine and glycine de novo synthesis pathway in PANC-1 cells, we assembled lentiviral short hairpin RNA (shRNA) vectors targeting *PHGDH* and/or *SHMT1* genes to interfere the metabolic flux (Additional file 1: Figure S2A). We inoculated the PHGDH knockdown cells into the nude mice. Clearly, PHGDH knockdown significantly impaired the tumor growth (Fig. 2a). Orthotopic xenograft model showed that PHGDH knockdown reduced the metastases of primary tumor to liver and lung (Fig. 2b and c) and significantly prolonged the survival of tumor bearing nude mice compared with control (Fig. 2d). Combining with clinical data, we analyzed the mRNA expression levels of PHGDH and SHMT1 in clinical samples from The Cancer Genome Atlas (TCGA) pancreatic adenocarcinoma cohort [29, 30]. The patients with low PHGDH mRNA expression (z score < − 0.5) showed better prognosis than others and so did the patients with low PHGDH and low SHMT1 mRNAs expression (Fig. 2e and f). However, the expression of SHMT1 was not as substantial as that of PHGDH (data not shown). The above results demonstrate that PHGDH dominantly controlled this synthesis pathway and positively related with pancreatic cancer development.

In the in vitro studies, PHGDH inhibitor CBR5884 [22], which inhibited serine synthesis in a dose dependent manner, significantly suppressed the proliferation of PANC-1 cells (Additional file 1: Figure S2B and C). Cisplatin and paclitaxel, generally used chemotherapeutics to treat advanced pancreatic cancer [31, 32], also inhibited PANC-1 cells proliferation in a dose dependent manner (Additional file 1: Figure S2D and E). Respectively combining with PHGDH inhibitor, the cytotoxicity of cisplatin and paclitaxel were obviously enhanced compared with cisplatin and paclitaxel alone. And their triple combinations achieved the maximum cytotoxicity, which decreased the viability of PANC-1 cells nearly by 90% (Fig. 2g). The combination of PHGDH inhibitor and chemotherapeutics provides a prospective therapeutic strategy to treat advanced pancreatic cancer.

Subsequently we detected PHGDH knockdown significantly reduced the total and labeled serine and glycine contents of PANC-1 cells, but SHMT1 knockdown only reduced the glycine contents (Additional file 1: Figure S2F and G), confirming that PHGDH exhibited a dominant role in serine and glycine de novo synthesis pathway. Similar to the effect of PHGDH inhibitor, PHGDH knockdown inhibited the cell proliferation and further caused apoptosis of PANC-1 cells, while had no effect on the cell cycle (Fig. 2h and Additional file 1: Figure S2H and I). Reversely, serine supplements rescued the proliferation of PHGDH knockdown cells in a dose dependent manner (Additional file 1: Figure S2J). However, serine supplements did not completely compensate for the PHGDH knockdown (Fig. 2i and j). These results indicated that the reduction of serine contents was only a partial reason for the decreased proliferation of PHGDH knockdown cells.

Subsequently, we explored the regulative effect of PHGDH on tumorigenesis by means of colony formation assay. Under normal conditions, the colony formations were disrupted by PHGDH knockdown (Fig. 2k). Similar to the results of proliferation assays, serine supplements only partially increased the colony number but did not completely rescue the PHGDH knockdown (Fig. 2l).

From the above results, we found no significant difference between PHGDH single knockdown and PHGDH/SHMT1 double knockdowns in cell proliferation and colony formation (Fig. 2h and k). The rescuing effect of serine on cell proliferation was more significant than glycine and was equivalent with their mixture (Fig. 2i and j). These results further indicate that PHGDH dominantly controls the serine and glycine synthesis pathway. In addition, serine supplies insufficient to completely rescue the knockdown of PHGDH implies that besides catalyzing serine synthesis, some unknown functions of PHGDH also participate in regulating cell proliferation and tumorigenesis.

### PHGDH regulates cell-cell tight junction-related proteins expression

**Fig. 3:**
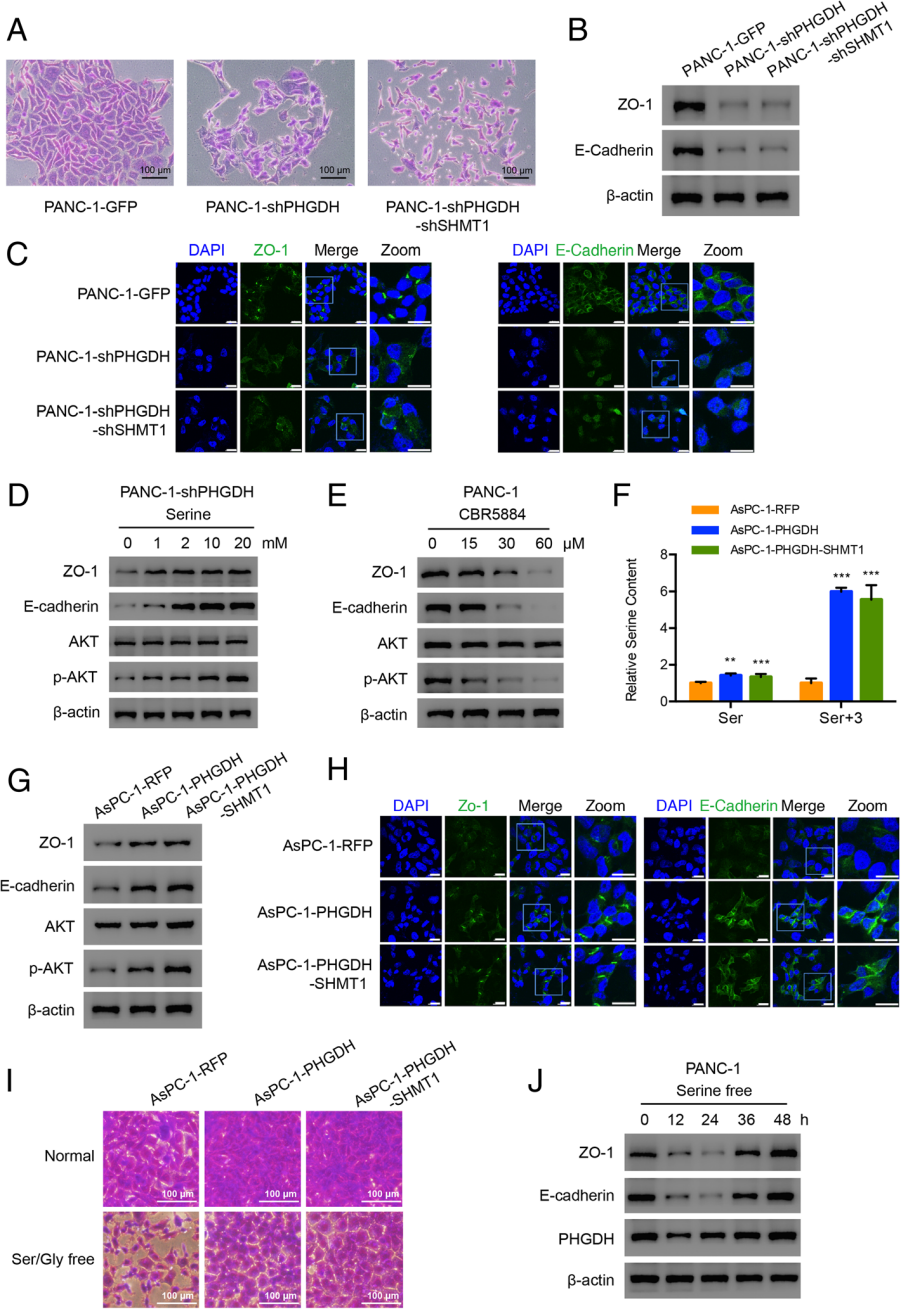
PHGDH regulates cell-cell tight junction-related proteins expression. **a** Enlarged single colony images of the constructed PANC-1 cells in the colony formation assays. Scale bar, 100 μm. **b** Western blot and (**c**) Immunofluorescence results of the tight junction-related proteins in the constructed PANC-1 cells. Scale bar, 20 μm. **d** Western blot analysis of the indicated protein expression levels in PANC-1-shPHGDH cells under the treatment of 0, 1, 2, 10, 20 mM serine. **e** Western blot analysis of the indicated protein levels in the PANC-1 cells treated by CBR5884 for 12 h. **f** Relative total and labeled serine contents in the constructed AsPC-1 cells. Data are representative of at least three independent experiments. p value: Student’s t-test; **p < 0.01, ***p < 0.001. Columns: mean; bars: SD. **g** Western blot analysis of the indicated protein levels in the constructed AsPC-1 cells. **h** Immunofluorescence results of the indicated proteins in the constructed AsPC-1 cells. Scale bar, 20 μm. **i** Enlarged single colony images of the constructed AsPC-1 cells in the colony formation assays. Scale bar, 100 μm. **j** Western blot analysis of the indicated proteins in the PANC-1 cells treated by serine free condition

In the colony formation assays, PHGDH knockdown not only significantly reduced the colony numbers (Fig. 2k) but also dramatically damaged the cell-cell tight junctions in single colony (Fig. 3a). The expression of tight junction-related proteins including ZO-1 and E-cadherin [33] were significantly decreased in PHGDH knockdown cells (Fig. 3b). Their membrane localizations were correspondingly decreased (Fig. 3c). When we supplied PHGDH knockdown cells with serine to restore the intracellular serine contents (Additional file 1: Figure S3A), the levels of ZO-1, E-cadherin and phosphorylated AKT were gradually rescued in a dose dependent manner (Fig. 3d). The similar effects of serine were also found in PANC-1 cells (Additional file 1: Figure S3B). Conversely, we treated PANC-1 cells with the PHGDH inhibitor CBR5884 [22] and observed that along with serine reduction (Additional file 1: Figure S2B), the expression of ZO-1 and E-cadherin significantly decreased and so did the phosphorylated AKT level (Fig. 3e). These results indicated that PHGDH knockdown resulted in decreases of serine and AKT activity, which attenuated the expression of ZO-1 and E-cadherin. To further confirm PHGDH promoted the relevant proteins expression, it was overexpressed in PHGDH low expressing AsPC-1 cells (Additional file 1: Figure S3C and D). Along with the increases of serine and glycine contents (Fig. 3f and Additional file 1: Figure S3E), the expression of ZO-1 and E-cadherin and their membrane localizations were all promoted by the overexpression of PHGDH (Fig. 3g and h). The tight junctions between cells became more intensive, especially under serine free conditions (Fig. 3i). Therefore, the cell proliferations were significantly increased (Additional file 1: Figure S3F). However, when PANC-1 cells were cultured in serine free media for total 48 h, along with serine continual consumption (Additional file 1: Figure S3G), the levels of ZO-1 and E-cadherin reduced in the initial 24 h but increased subsequently, in parallel with the increased PHGDH expression, which was supposed to overcome the serine starvation (Fig. 3j). This result implied that PHGDH expression was also involved in regulating the proteins expression of ZO-1 and E-cadherin.

### PHGDH interacts with eIF4A1 and eIF4E in PANC-1 cells

**Fig. 4:**
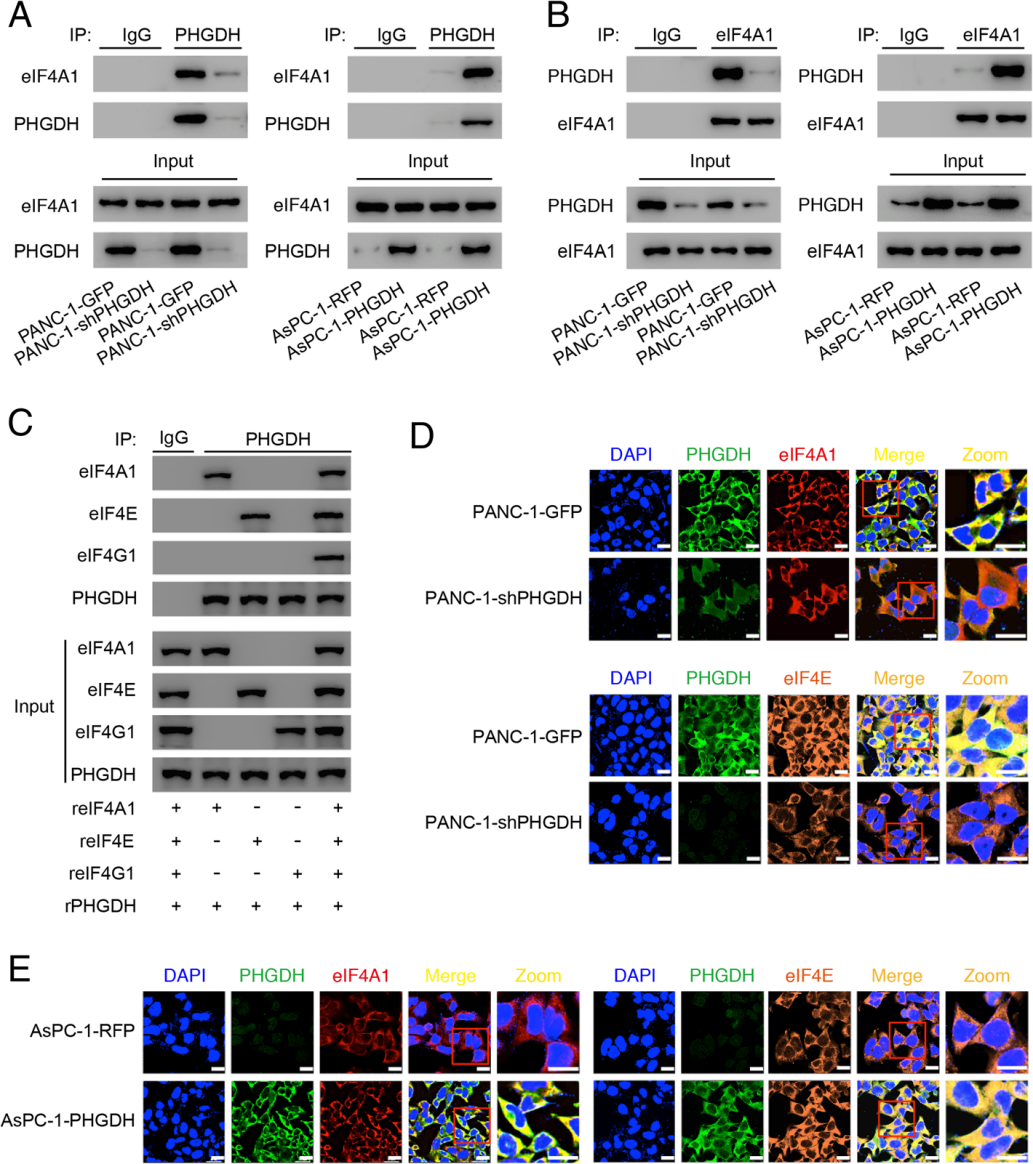
PHGDH interacts with eIF4A1 and eIF4E in PANC-1 cells. **a** Immunoprecipitation results of the interaction between PHGDH (bait) and eIF4A1, in PANC-1-GFP and PANC-1-shPHGDH cells (left), AsPC-1-RFP and AsPC-1-PHGDH cells (right). **b** Reverse immunoprecipitation results of the interaction between PHGDH and eIF4A1 (bait). **c** Pull down assay results of the interactions between recombinant PHGDH (bait) protein, recombinant eIF4A1 protein, recombinant eIF4E protein and recombinant eIFG1 protein. “r” shorted for recombinant. **d** Immunofluorescence results of the overlapping signals between PHGDH and eIF4A1 or eIF4E in PANC-1-GFP and PANC-1-shPHGDH cells. Scale bar, 20 μm. **e** Immunofluorescence results of the overlapping signals between PHGDH and eIF4A1 or eIF4E in AsPC-1-RFP and AsPC-1-PHGDH cells. Scale bar, 20 μm

To investigate the non-enzymatic function of PHGDH, we explored whether PHGDH interacted with some proteins to regulate pancreatic cancer cells behavior. By means of co-immunoprecipitation and mass-spectrometry methods (Additional file 1: Figure S4A and B and Additional file 2: Table S1), PHGDH was found to interact with the eukaryotic translation initiation factor 4A1 (eIF4A1) (Fig. 4a and b). EIF4A is one of the three subunits in eIF4F complex, which also contains eIF4E and eIF4G. In the translation initiation process, eIF4E recognizes the m^7^-GTP cap on mRNA and recruits eIF4G [34, 35]. EIF4G, a large scaffolding protein involved in recruiting the 43S pre-initiation complex [36, 37], directly binds with eIF4A [38]. EIF4A is a DEAD-box RNA helicase implicated in preparing a ribosome landing pad for 43S pre-initiation complexes by unwinding the 5’ mRNA structure [39]. In mammals, there are two highly related eIF4A homologs, eIF4A1 and eIF4A2, eIF4A1 is generally the more abundantly expressed one [40–42]. Knockdown of PHGDH, which did not crucially affect the level of eIF4A1 (Additional file 1: Figure S4C), significantly reduced the interaction (Fig. 4a and b). Correspondingly, overexpression of PHGDH strongly augmented this interaction (Fig. 4a and b).

To further detect and confirm the interactions, we used recombinant proteins of PHGDH, eIF4A1, eIF4E and eIF4G1 to perform in vitro pull down assays. Results showed that PHGDH not only directly interacted with eIF4A1 but also interacted with eIF4E (Fig. 4c). While, PHGDH did not directly interact with eIF4G1, it could assemble with eIF4G1 through interacting with eIF4A1 and eIF4E (Fig. 4c). The immunofluorescence assay visualized the overlapping signals between PHGDH and eIF4A1 or eIF4E in the PANC-1-GFP cells, however, the overlap was receded after PHGDH knockdown (Fig. 4d and Additional file 1: Figure S4D). In PHGDH low expressing AsPC-1-RFP cells, the overlapping signals between PHGDH and eIF4A1 or eIF4E were very weak, but in PHGDH overexpressing AsPC-1 cells, PHGDH closely interacted with eIF4A1 and eIF4E (Fig. 4e and Additional file 1: Figure S4D).

### Knockdown of PHGDH inhibits the translation initiation by suppressing the activities of translation initiation factors and disassembling eIF4F complex

**Fig. 5:**
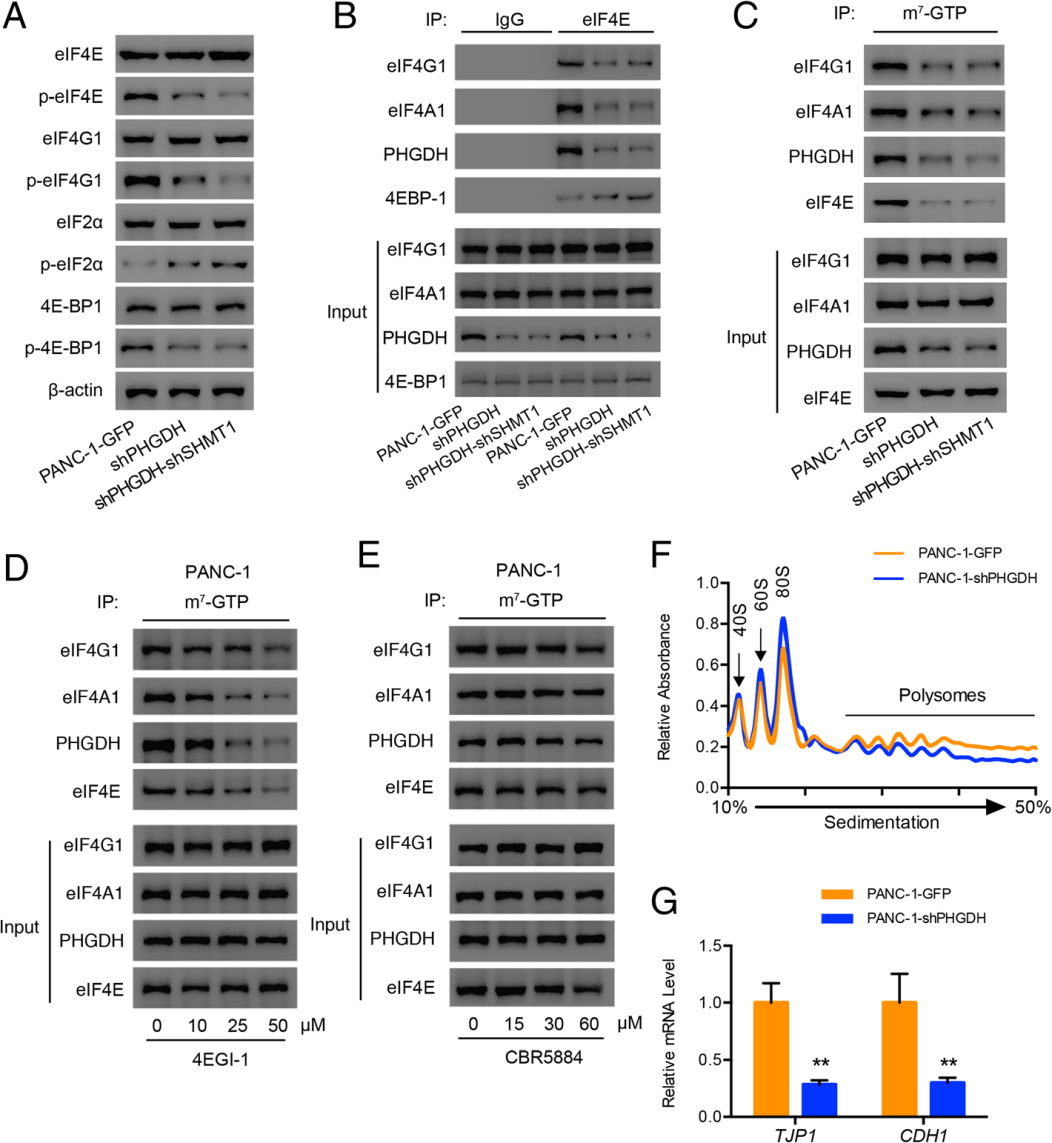
Knockdown of PHGDH inhibits the translation initiation by suppressing the activities of translation initiation factors and disassembling eIF4F complex. **a** Western blot analysis of the phosphorylation of translation initiation related factors eIF4E, eIF4G1, eIF2α and 4E-BP1 in the constructed PANC-1 cells. **b** Co-immnoprecipitation results of eIF4G1, eIF4A1, PHGDH and 4E-BP1 pulled down by eIF4E in the constructed PANC-1 cells. **c** m^7^-GTP pull-down detection of the translation initiation complex formation in the constructed PANC-1 cells. **d** and **e** m^7^-GTP pull-down detection of the translation initiation complex formation under the treatments of 4EGI-1 and CBR5884 in PANC-1 cells. **f** Polysomes profiling of PANC-1-GFP and PANC-1-shPHGDH cells treated with 100 mg/ml cycloheximide for 10 min. Absorbance light at 254 nm. **g** Extracted the polysomes associated mRNA of PANC-1-GFP and PANC-1-shPHGDH cells and detected the ZO-1 and E-cadherin mRNA levels using qRT-PCR method. Data are representative of at least three independent experiments. p value: Student’s t-test; **p < 0.01. Columns: mean; bars: SD

Translation of most mRNAs is controlled at the rate-limiting steps. First, the 43S ribosome pre-initiation complex formation, consists of the 40S small ribosomal subunit, the initiating methionyl tRNA (Met-tRNAi) and a group of eukaryotic initiation factors (eIFs), such as eIF2. The activity of the 43S pre-initiation complex is controlled by the phosphorylation state of eIF2α. In GTP-bound state, eIF2-Met-tRNAi complex allows 43S ribosomes to initiate translation, while, when eIF2α is phosphorylated, the recycling of GTP on eIF2α is blocked thereby inhibiting protein synthesis [38]. Next, the 43S ribosome complex is recruited to the 5′ end of the mRNA binding with eIF4G. Increased phosphorylation of eIF4G is associated with increased protein synthesis [43, 44]. EIF4G combines with eIF4E and facilitates its phosphorylation by bringing it into proximity with the eIF4G-bound MNK1 and MNK2 kinases [45]. It is reported that eIF4E phosphorylation acts a positive role in promoting cancer progression [46]. We detected the phosphorylation of these translation factors eIF4E, eIF4G1, eIF2α and the blocker 4E-BP1 in PHGDH knockdown cells. Figure 5a showed that PHGDH knockdown reduced the phosphorylation of eIF4E, eIF4G1, 4E-BP1 and increased the phosphorylation of eIF2α, which implied that the translation initiation was inhibited.

Next we detected the integrity of translation initiation complex and its assembly on the mRNA 5′-cap structure. In the absence of mitogenic signaling, inhibitory 4E binding proteins (4EBPs) bind with and sequester eIF4E from interaction with eIF4G [34, 35]. The co-immunoprecipitation results showed that in the PANC-1-GFP cells, eIF4G1, eIF4A1 and eIF4E assembled together to form the complete eIF4F complex, however, in the PHGDH knockdown cells, the eIF4F complex was disassembled and more eIF4E were blocked by 4E-BP1 (Fig. 5b). Analog of the mRNA 5′-cap structure, m^7^-GTP, was used to determine the translation initiation status. Results showed that knockdown of PHGDH in PANC-1 cells disrupted the assembly of eIF4F complex on m^7^-GTP (Fig. 5c), similar to the effect of 4EGI-1 treatments (Fig. 5d), which impaired the cell viability in dose and time dependent manners (Additional file 1: Figure S5A and B). While, when we treated PANC-1 cells with PHGDH inhibitor CBR5884, the assembly of eIF4F complex on m^7^-GTP was not affected (Fig. 5e), demonstrating that this process was independent of serine. By comparing the levels of translational active polysomes between the PANC-1-GFP cells and the PHGDH knockdown cells, we observed that PHGDH knockdown decreased the level of polysomes (Fig. 5f), and the polysomes associated mRNA levels of ZO-1 and E-cadherin (Fig. 5g).

The above results demonstrated that knockdown of PHGDH suppressed the activities of translation initiation factors and disassembled the eIF4F complex, thus finally inhibited the translation initiations of the relevant proteins.

### Overexpression of PHGDH enhances translation initiation to further promote the pancreatic cancer development

**Fig. 6:**
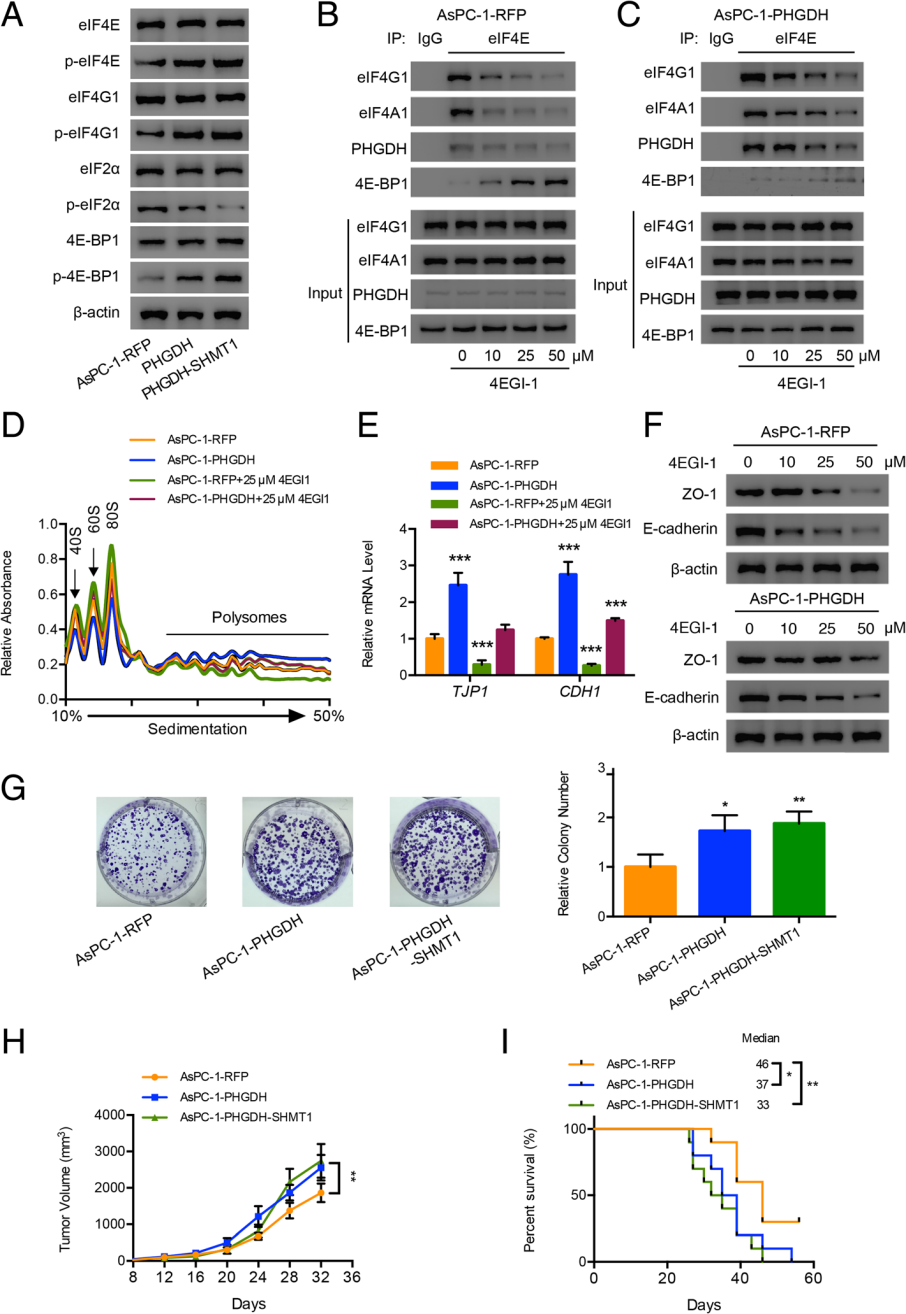
Overexpression of PHGDH enhances translation initiation to further promote the pancreatic cancer development. **a** Western blot analysis of the phosphorylation of translation initiation related factors eIF4E, eIF4G1, eIF2α and 4E-BP1 in the constructed AsPC-1 cells. **b** and **c** Co-immnoprecipitation results of the integrity of translation initiation complex eIF4F in AsPC-1-RFP and AsPC-1-PHGDH cells under the treatment of 4EGI-1 for 12 h. **d** Polysomes profiling of AsPC-1-RFP and AsPC-1-PHGDH cells treated by 4EGI-1 for 12 h. **e** Extracted the polysomes associated mRNA of AsPC-1-RFP cells and AsPC-1-PHGDH cells treated by 4EGI-1 for 12 h and detected the ZO-1 and E-cadherin mRNA levels using qRT-PCR method. **f** Western blot analysis of the indicated protein levels under the treatment of 4EGI-1 in AsPC-1-RFP and AsPC-1-PHGDH cells. **g** Colony formation results of the constructed AsPC-1 cells under the normal conditions. **h** Results of animal experiments. The constructed AsPC-1 cells were inoculated subcutaneously into the nude mice. Tumor sizes were measured every 3 or 4 days. Each value is the mean ± SEM of determinations in at least 6 nude mice of each group. **i** Results of animal experiments. The constructed AsPC-1 cells were inoculated orthotopically into the nude mice. The survival statuses were analyzed for 8 weeks. Each group at least contained 6 nude mice. Data are representative of at least three independent experiments. p value: Student’s t-test; *p < 0.05,**p < 0.01. Columns: mean; bars: SD

In AsPC-1 cells, PHGDH overexpression converted the translation factors to more active status, implying that the translation initiation was enhanced (Fig. 6a). 4EGI-1 is an inhibitor of eIF4F complex that prevents the cap-dependent translation [38, 47]. We treated AsPC-1-RFP cells with low-toxic doses of 4EGI-1 (Additional file 1: Figure S6A and B) and observed the assembly of eIF4G1, eIF4A1 and eIF4E was easily disrupted, and more eIF4E were blocked by 4E-BP1 along with the doses increase of 4EGI-1 (Fig. 6b). In PHGDH overexpressing AsPC-1 cells, even under the treatments of 4EGI-1, eIF4G1, eIF4A1 and eIF4E still combined together and little eIF4E was blocked by 4EBP-1 (Fig. 6c). In addition, 4EGI-1 treatments did not affect the serine contents in the AsPC-1-RFP and AsPC-1-PHGDH cells (Additional file 1: Figure S6C and D), indicating that the integrity of eIF4F was also independent of serine content. Furthermore, overexpression of PHGDH in AsPC-1 cells elevated the polysomes level (Fig. 6d), as well as the polysomes associated mRNA levels of ZO-1 and E-cadherin (Fig. 6e). 4EGI-1 treatments obviously inhibited the active translation initiation in AsPC-1-RFP cells but PHGDH overexpression significantly relieved the inhibition (Fig. 6d and e). Since the overexpressed PHGDH helped to maintain the formation of eIF4F and the translational active mRNA level under 4EGI-1 interruptions, the expression of ZO-1 and E-cadherin were still sustained to higher levels compared with that of AsPC-1-RFP cells (Fig. 6f). Finally, PHGDH overexpression significantly enhanced the tumorigenesis ability of AsPC-1 cells (Fig. 6g). The results of in vivo studies further identified that PHGDH overexpression converted the tumor to a more highly developing state (Fig. 6h) and the overall survival of orthotopic xenograft mouse model was further shortened (Fig. 6i).

## Discussion

Glucose is one of the most essential nutrients exploited for mitochondrial oxidative phosphorylation to generate energy needed for cellular behaviors in normal differentiated cells. While, most cancer cells, instead, exploit it for aerobic glycolysis, which is called “the Warburg effect” [5]. Aerobic glycolysis is an inefficient way to generate ATP, but in cancer cells or even all highly proliferating cells, it is adapted to facilitate the uptake and incorporation of nutrients into the biomass (e.g., nucleotides, amino acids and lipids) needed to produce a new cell [6]. Cancer-associated mutations enable cancer cells to acquire and metabolize nutrients in a manner conducive to proliferation rather than efficient ATP production [6, 48]. In current study, it is identified the largely consumed glucose in glycolysis process is highly diverted into serine and glycine synthesis in PANC-1 cells through catalysis of the abundantly expressed PHGDH and SHMT1. While in breast cancer cells MDA-MB-231, there is no amplification of PHGDH [11], the highly consumed glucose is diverted into alanine but not serine or glycine synthesis. These results indicate that different types of cancer cells show different metabolic preferences in aerobic glycolysis.

Knockdown of PHGDH significantly reduces the serine and glycine contents in PANC-1 cells and inhibits cell proliferation and tumorigenesis through impairing the cell-cell tight junctions. Serine supplement can significantly rescue the cell proliferation impaired by PHGDH knockdown but glycine can not. Serine and glycine can be inter-converted by the cytoplasmic SHMT1 [10, 16] and the mitochondria SHMT2 [16, 49, 50]. The conversion of serine to glycine generates one-carbon units which can be the substrate for nucleotide synthesis to promote cell proliferation [51]. While, single glycine supplement can not provide enough one-carbon units to support purine synthesis. Otherwise, excess glycine supplement reversely consumes one-carbon units for serine synthesis that blocks the purine synthesis and inhibits the cell proliferation [52]. Therefore, in PHGDH knockdown cells, the promotion effect of serine on cell proliferation is significant but glycine is not.

We also construct the SHMT1 knockdown cells. Suppression of SHMT1 impairs the cell proliferation and colony formation (Additional file 1: Figure S7A and B). But reversely, serine or glycine supplement completely rescues SHMT1 knockdown (Additional file 1: Figure S7C and D). These results indicate that the major function of cytosolic SHMT1 is catalyzing glycine synthesis. However, in PHGDH knockdown cells, serine supplement can not completely rescue the cell proliferation, suggesting that the tumor-promoting function of PHGDH is not limited to amino acid synthesis.

In cap-dependent translation initiation process, activation of the PI3K/AKT/mTOR signaling pathway liberates eIF4E from 4E-BP1 [53], otherwise, eIF4E is blocked by 4E-BP1 from interaction with eIF4G [34, 35]. Under serine starvation condition, continuous serine reduction inhibits the activation of AKT, however, the highly expressed PHGDH still promotes the expression of ZO-1 and E-cadherin. Results show that PHGDH overexpression can potently sustain the interaction of eIF4E with eIF4G and meanwhile reduce the blockage of eIF4E by 4E-BP1 under the treatment of 4EGI-1. Therefore, we suppose that, under serine starvation condition, the highly expressed PHGDH can sustain the interaction of eIF4E with eIF4G and avoid the blockage of 4E-BP1. The translation initiation complex eIF4F can still bind to the 5’ mRNA structure to initiate translation. Once the translation being started, eIF4G/eIF4A dimers will be sufficient to sustain multiple subsequent rounds of initiation to initiate the protein expression [42].

PHGDH is found to directly interact with eIF4A1 and eIF4E. In the following studies, we will explore the exact binding positions of PHGDH/eIF4A1 and PHGDH/eIF4E. It is reported that the di-nucleotide binding domain of PHGDH, which is close to its substrate-binding pockets, serves for RNA binding [25, 26]. Since eIF4A1 and eIF4E combine with 5’ mRNA structure, we speculate that the interacting positions of PHGDH with eIF4A1 and eIF4E relate to its di-nucleotide binding domain, and may be also close to its substrate-binding pockets.

Pancreatic cancer is hard to treat because of its strong metastatic ability [3]. Although PHGDH knockdown decreases the E-cadherin expression and its decrease is an important step of EMT to facilitate tumor metastasis [54], in this study, PHGDH knockdown inhibits the tumor metastasis. This phenomenon is also reported in another paper, in which heterozygous loss of Dpc4 down-regulates E-cadherin expression but significantly impairs the cells migration and invasion [55]. And also, we detect other EMT markers, such as N-cadherin and β-catenin. Their expressions are not significantly changed by PHGDH knockdown (data not shown). Based on the above results we infer that PHGDH regulates the metastasis of pancreatic cancer not through the EMT mechanism. According to our latest data, PHGDH expression regulates the cross talk and chemokine expression in pancreatic cancer cells and fibroblast in the tumor microenvironment, which facilitates cancer cells invasion and distribution to distant organs. And its function in remodeling the extracellular matrix also benefits for cancer cells extravasation.

## Conclusion

This study reveals that PHGDH, the enzyme of glycolysis branching pathway, not only provides the essential compounds serine and glycine, but also directly participates in controlling cell behaviors. This is the first time that PHGDH is proved to possess the regulatory function in translation initiation through interacting with eIF4A1 and eIF4E to directly regulate the relevant proteins expression. The systematic studies from the cellular level to the animal experiments and further to the clinical patients analysis convincingly demonstrate the importance of PHGDH in pancreatic cancer development. Inhibiting PHGDH expression and disrupting the interactions between PHGDH and eIF4A1/eIF4E provide new prospects for anti-tumor therapeutics designs, which are meaningful for improving the pancreatic cancer treatment effect and prolonging the overall survival of clinical patients.

## Additional files

Additional file 1: **Figure S1.** Detection of pyruvate concentrations in different cell types. **Figure S2.** Reduction of serine caused by PHGDH knockdown induces apoptosis and inhibits cell proliferation. **Figure S3.** Serine supply promotes the indicated proteins expression; PHGDH overexpression increases the intracellular glycine contents and promotes the proliferation of AsPC-1 cells. **Figure S4.** PHGDH interacts with eIF4A1 and eIF4E. **Figure S5.** Cytotoxicity test of 4EGI-1 in PANC-1 cells. **Figure S6.** Cytotoxicity test of 4EGI-1 and its effect on serine contents in the constructed AsPC-1 cells. **Figure S7.** Serine or glycine supplement completely rescues the impaired cell proliferation and colony formation caused by SHMT1 knockdown. (DOCX 2438 kb)

Additional file 2: **Table S1.** Immunopercipitation and mass-spectrometry results of PHGDH interacting proteins. (DOCX 60 kb)

## Abbreviations

4E-BPs: Eukaryotic translation initiation factor 4E-binding proteins
eIF: Eukaryotic translation initiation factor
m^7^-GTP: 7-Methyl-GTP
PHGDH: Phosphoglycerate dehydrogenase
SHMT1: Serine hydroxymethyltransferase 1
TCA: Tricarboxylic acid
TCGA: The Cancer Genome Atlas
