# Correction: Phosphoglycerate dehydrogenase promotes pancreatic cancer development by interacting with eIF4A1 and eIF4E

**DOI:** 10.1186/s13046-024-03252-z

**Published:** 2024-12-23

**Authors:** Xuhui Ma, Boya Li, Jie Liu, Yan Fu, Yongzhang Luo

**Affiliations:** 1 The National Engineering Laboratory for Anti-Tumor Protein Therapeutics, Tsinghua University, Beijing, 100084 China; 2 Beijing Key Laboratory for Protein Therapeutics, Tsinghua University, Beijing, 100084 China; 3 Cancer Biology Laboratory, School of Life Sciences, Tsinghua University, Beijing, 100084 China

**Correction** **:** **J Exp Clin Cancer Res 38, 66 (2019)**

**https://doi.org/10.1186/s13046-019-1053-y**

Following publication of the original article [1], the authors identified errors in the figures and the Supplementary Material, specifically:

- Figure 2C - metastasis foci image of the PANC-1-shPHGDH-shSHMT1 group was incorrectly used
- Figure 2K, 2L and Supplementary Material S7D - colony formation images were incorrectly used
- Figure 3G - western blot figures were incorrectly used

The correct figures are given below.

**Incorrect Fig. 2**


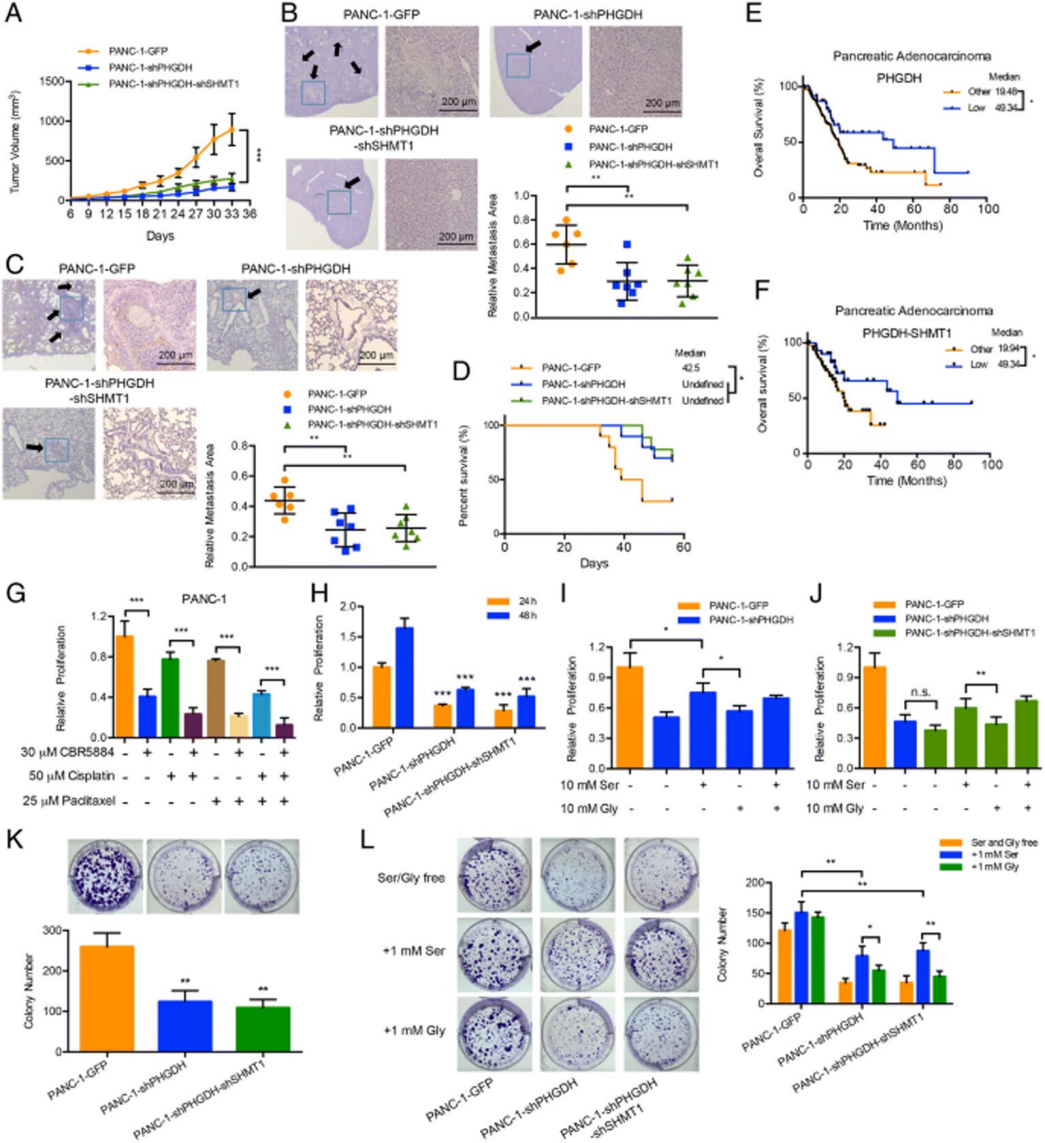


**Fig. 2** Knockdown of PHGDH attenuates pancreatic cancer development through inhibiting cell proliferation and tumorigenesis. **a** Nude mice were subcutaneously inoculated with the constructed PANC-1 cells. Tumor sizes were measured every 3 or 4 days. Each value is the mean ± SEM of determinations in at least 6 nude mice of each group. **b, c** and **d** Nude mice were orthotopically inoculated with the constructed PANC-1 cells. **b** and **c** Liver and lung metastases (arrows, metastatic foci) were detected with HE staining. Scale bars, 200 μm. The quantitative results represented the relative area of metastatic foci to the whole tissue slide; **d** The survival statuses were analyzed for 8 weeks. Each group at least contained 6 nude mice. **e** and **f** Kaplan-Meier curve for overall survival of the pancreatic adenocarcinoma patients based on (**e**) PHGDH mRNA and (**f**) PHGDH and SHMT1 mRNAs expression in the TCGA dataset. The blue line represented the patients with (**e**) low PHGDH and (**f**) low PHGDH and low SHMT1 expression levels (z-score < − 0.5); the black line represented other patients (z-score ≥ − 0.5). **e** *n* = 178. **f** *n* = 80. **g** Relative proliferation results of PANC-1 cells under the treatments of 30 μM PHGDH inhibitor CBR5884, 50 μM cisplatin and/or 25 μM paclitaxel for 24 h. **h** Relative proliferation results of the constructed PANC-1 cells for 24 and 48-h cultures. **i** and **j** Relative proliferation results of the constructed PANC-1 cells under the treatments of 10 mM serine and/or 10 mM glycine.** k** and **l** Colony formation results of the constructed PANC-1 cells under (**k**) the normal conditions and (**l**) the conditions of serine and glycine free (up), 1 mM serine supply (middle) and 1 mM glycine supply (down) respectively. Data are representative of at least three independent experiments. *p* value: Student’s t-test; *p < 0.05,**p < 0.01, ***p < 0.001. Columns: mean; bars: SD

**Correct Fig. 2**


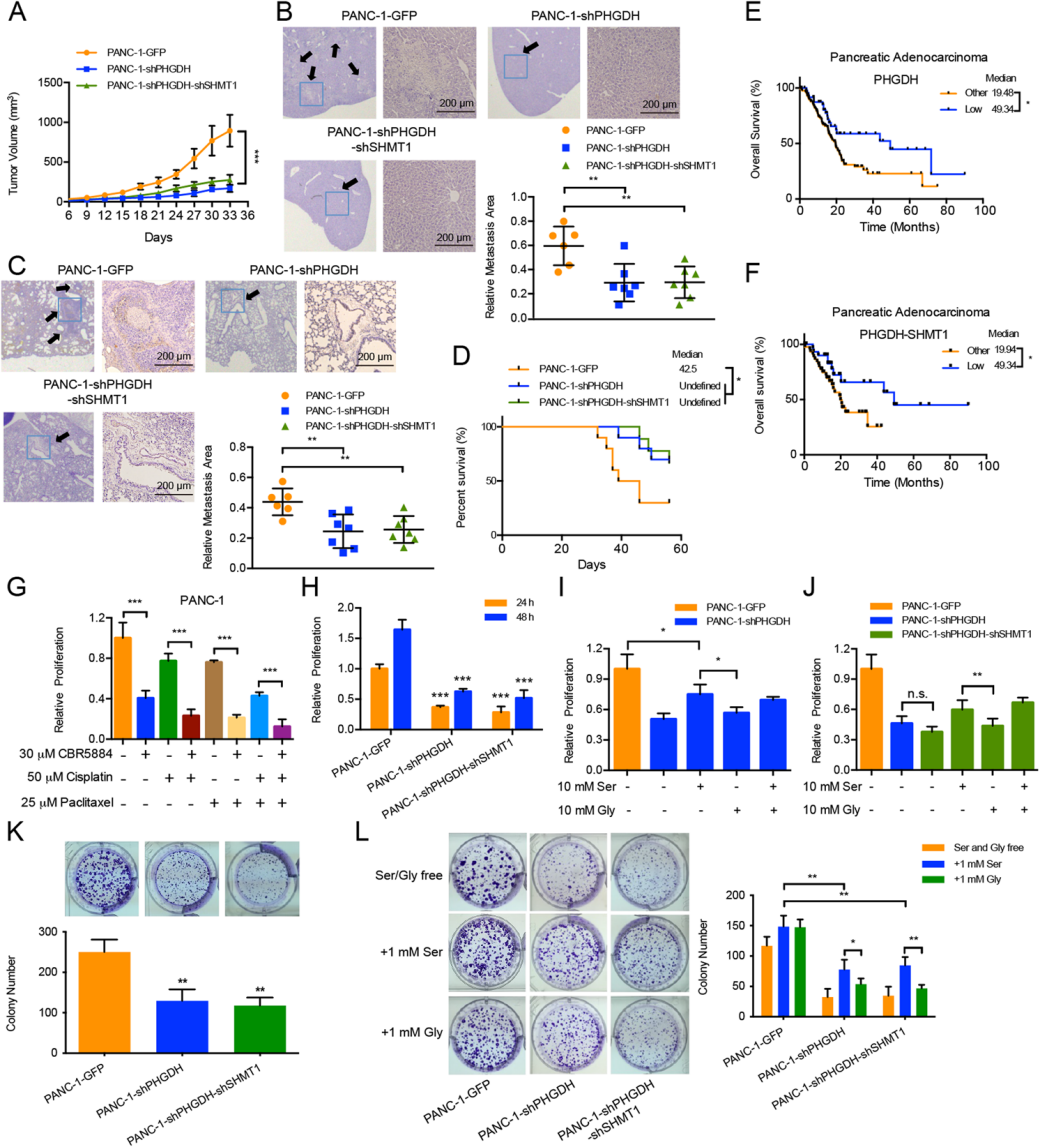


**Fig. 2** Knockdown of PHGDH attenuates pancreatic cancer development through inhibiting cell proliferation and tumorigenesis. **a** Nude mice were subcutaneously inoculated with the constructed PANC-1 cells. Tumor sizes were measured every 3 or 4 days. Each value is the mean ± SEM of determinations in at least 6 nude mice of each group. **b, c** and **d** Nude mice were orthotopically inoculated with the constructed PANC-1 cells. **b** and **c** Liver and lung metastases (arrows, metastatic foci) were detected with HE staining. Scale bars, 200 μm. The quantitative results represented the relative area of metastatic foci to the whole tissue slide; **d** The survival statuses were analyzed for 8 weeks. Each group at least contained 6 nude mice. **e** and **f** Kaplan-Meier curve for overall survival of the pancreatic adenocarcinoma patients based on **e** PHGDH mRNA and **f** PHGDH and SHMT1 mRNAs expression in the TCGA dataset. The blue line represented the patients with **e** low PHGDH and **f** low PHGDH and low SHMT1 expression levels (z-score < − 0.5); the black line represented other patients (z-score ≥ − 0.5). **e** *n* = 178. **f** *n* = 80. **g **Relative proliferation results of PANC-1 cells under the treatments of 30 μM PHGDH inhibitor CBR5884, 50 μM cisplatin and/or 25 μM paclitaxel for 24 h. **h** Relative proliferation results of the constructed PANC-1 cells for 24 and 48-h cultures. **i **and **j** Relative proliferation results of the constructed PANC-1 cells under the treatments of 10 mM serine and/or 10 mM glycine. **k** and **l** Colony formation results of the constructed PANC-1 cells under **k** the normal conditions and **l** the conditions of serine and glycine free (up), 1 mM serine supply (middle) and 1 mM glycine supply (down) respectively. Data are representative of at least three independent experiments. *p* value: Student’s t-test; *p < 0.05,**p < 0.01, ***p < 0.001. Columns: mean; bars: SD

**Incorrect Fig. 3**


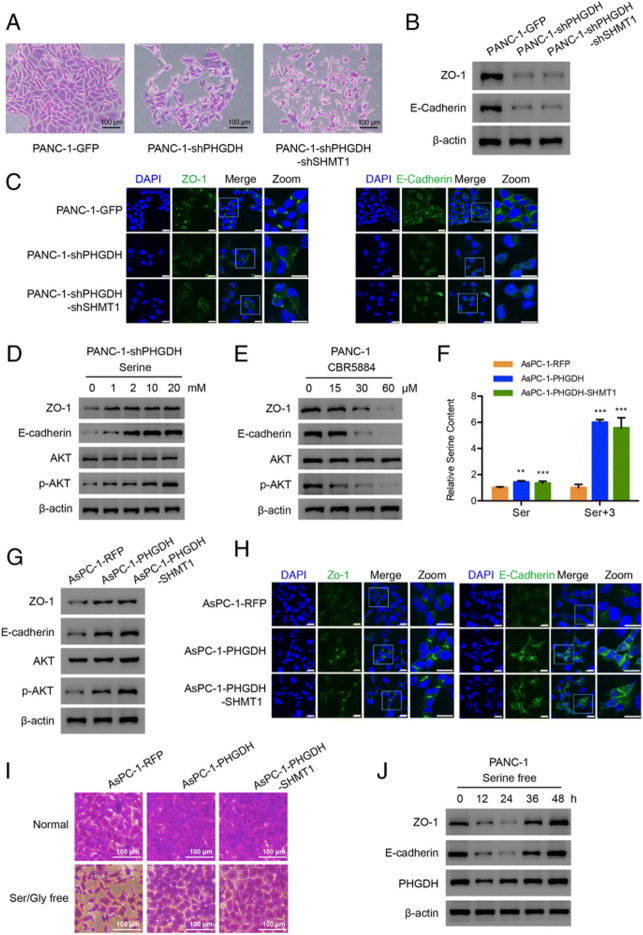


**Fig. 3** PHGDH regulates cell-cell tight junction-related proteins expression. **a** Enlarged single colony images of the constructed PANC-1 cells in the colony formation assays. Scale bar, 100 μm. **b** Western blot and (**c**) Immunofluorescence results of the tight junction-related proteins in the constructed PANC-1 cells. Scale bar, 20 μm. **d** Western blot analysis of the indicated protein expression levels in PANC-1-shPHGDH cells under the treatment of 0, 1, 2, 10, 20 mM serine. **e** Western blot analysis of the indicated protein levels in the PANC-1 cells treated by CBR5884 for 12 h. **f** Relative total and labeled serine contents in the constructed AsPC-1 cells. Data are representative of at least three independent experiments. p value: Student’s t-test; **p < 0.01, ***p < 0.001. Columns: mean; bars: SD. **g** Western blot analysis of the indicated protein levels in the constructed AsPC-1 cells. **h** Immunofluorescence results of the indicated proteins in the constructed AsPC-1 cells. Scale bar, 20 μm. **i** Enlarged single colony images of the constructed AsPC-1 cells in the colony formation assays. Scale bar, 100 μm. **j** Western blot analysis of the indicated proteins in the PANC-1 cells treated by serine free condition

**Correct Fig. 3**


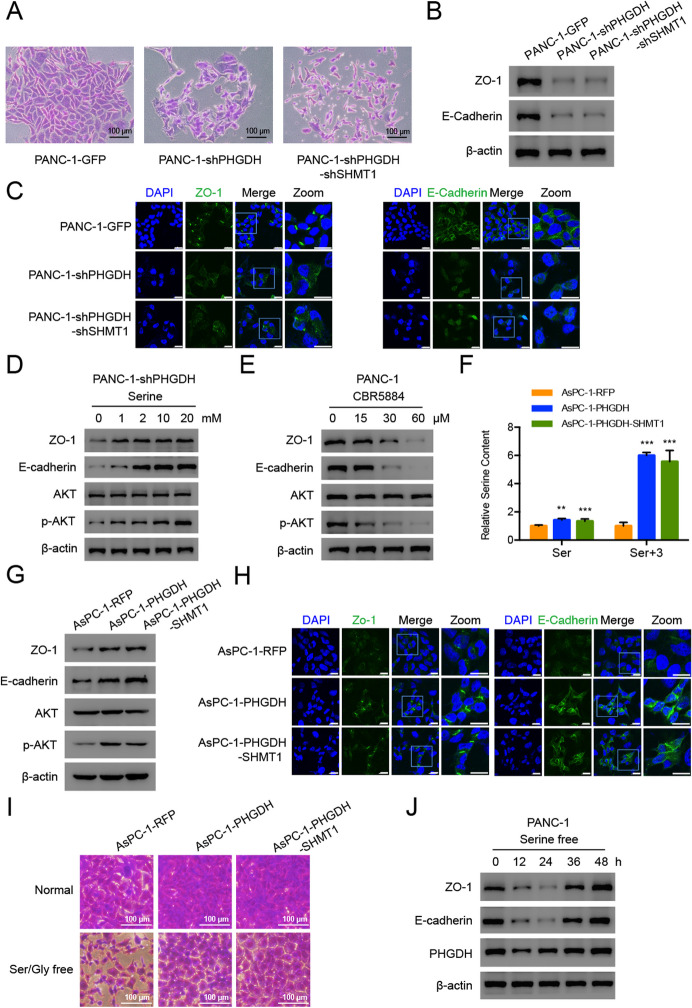


**Fig. 3** PHGDH regulates cell-cell tight junction-related proteins expression. **a** Enlarged single colony images of the constructed PANC-1 cells in the colony formation assays. Scale bar, 100 μm. **b** Western blot and **c** Immunofluorescence results of the tight junction-related proteins in the constructed PANC-1 cells. Scale bar, 20 μm. **d** Western blot analysis of the indicated protein expression levels in PANC-1-shPHGDH cells under the treatment of 0, 1, 2, 10, 20 mM serine. **e** Western blot analysis of the indicated protein levels in the PANC-1 cells treated by CBR5884 for 12 h. **f** Relative total and labeled serine contents in the constructed AsPC-1 cells. Data are representative of at least three independent experiments. p value: Student’s t-test; **p < 0.01, ***p < 0.001. Columns: mean; bars: SD. **g** Western blot analysis of the indicated protein levels in the constructed AsPC-1 cells. **h** Immunofluorescence results of the indicated proteins in the constructed AsPC-1 cells. Scale bar, 20 μm. **i** Enlarged single colony images of the constructed AsPC-1 cells in the colony formation assays. Scale bar, 100 μm. **j** Western blot analysis of the indicated proteins in the PANC-1 cells treated by serine free condition.

The correction does not have any effect on the conclusions of this article. The original article [1] has been updated.

## Supplementary Information

** Additional file 1:** **Figure S7.** Serine or glycine supplement completely rescues the impaired cell proliferation and colony formation caused by SHMT1 knockdown.
