# Supplementary material for: Phosphoglycerate dehydrogenase promotes pancreatic cancer development by interacting with eIF4A1 and eIF4E
Source: J Exp Clin Cancer Res. 2019 Feb 11;38:66. doi: 10.1186/s13046-019-1053-y (PMC6371491; doi:10.1186/s13046-019-1053-y)
Supplement: Supplementary file 2 — Table S1. Immunopercipitation and mass-spectrometry results of PHGDH interacting proteins. (DOCX 60 kb) [file 13046_2019_1053_MOESM2_ESM.docx]

**Table S1 Immunopercipitation and mass-spectrometry results of PHGDH interacting proteins**

| No. | Proteins | Score | Coverage |
| --- | --- | --- | --- |
| 1 | Eukaryotic initiation factor 4A1 | 32.10 | 16.50 |
| 2 | Elongation factor 1-alpha 1 | 26.03 | 20.13 |
| 3 | D-3-phosphoglycerate dehydrogenase | 22.80 | 9.57 |
| 4 | Heterogeneous nuclear ribonucleoprotein A/B | 22.44 | 13.21 |
| 5 | Elongation factor Tu, mitochondrial | 17.87 | 12.17 |
| 6 | Proliferation-associated protein 2G4 | 16.85 | 12.44 |
| 7 | 60S ribosomal protein L3 | 16.68 | 7.20 |
| 8 | Heterogeneous nuclear ribonucleoproteins C1/C2 (Fragment) | 16.07 | 34.85 |
| 9 | Heterogeneous nuclear ribonucleoprotein D0 (Fragment) | 15.76 | 21.62 |
| 10 | Leucine-rich repeat neuronal protein 4 | 12.23 | 2.70 |
| 11 | 6-phosphogluconate dehydrogenase, decarboxylating (Fragment) | 11.88 | 17.50 |
