# Supplementary material for: Correction: Phosphoglycerate dehydrogenase promotes pancreatic cancer development by interacting with eIF4A1 and eIF4E
Source: J Exp Clin Cancer Res. 2024 Dec 23;43:328. doi: 10.1186/s13046-024-03252-z (PMC11665198; doi:10.1186/s13046-024-03252-z)
Supplement: Supplementary file 1 — Additional file 1: Figure S7. Serine or glycine supplement completely rescues the impaired cell proliferation and colony formation caused by SHMT1 knockdown. [file 13046_2024_3252_MOESM1_ESM.docx]

**Figure S1.**


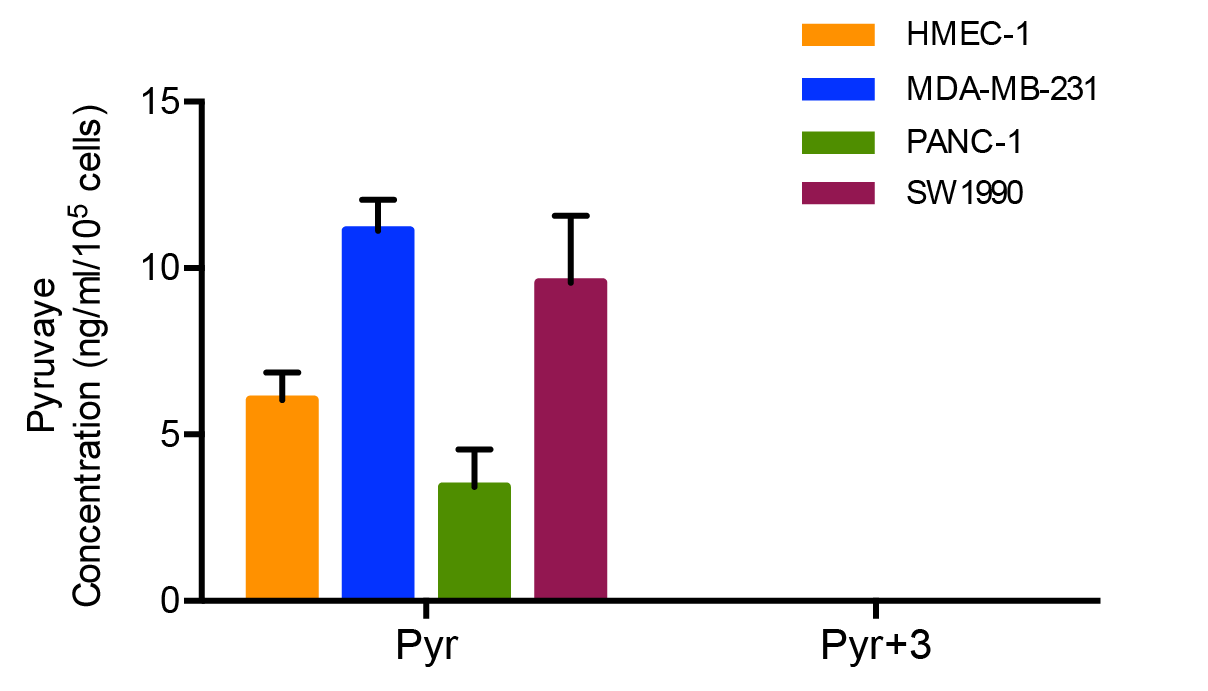


**Figure S1. Detection of pyruvate concentrations in different cell types.**

Carbon-unlabeled and labeled contents of pyruvate in HMEC, MDA-MB-231, PANC-1 and SW1990 cells. Data are representative of at least three independent experiments.

**Figure S2**

**
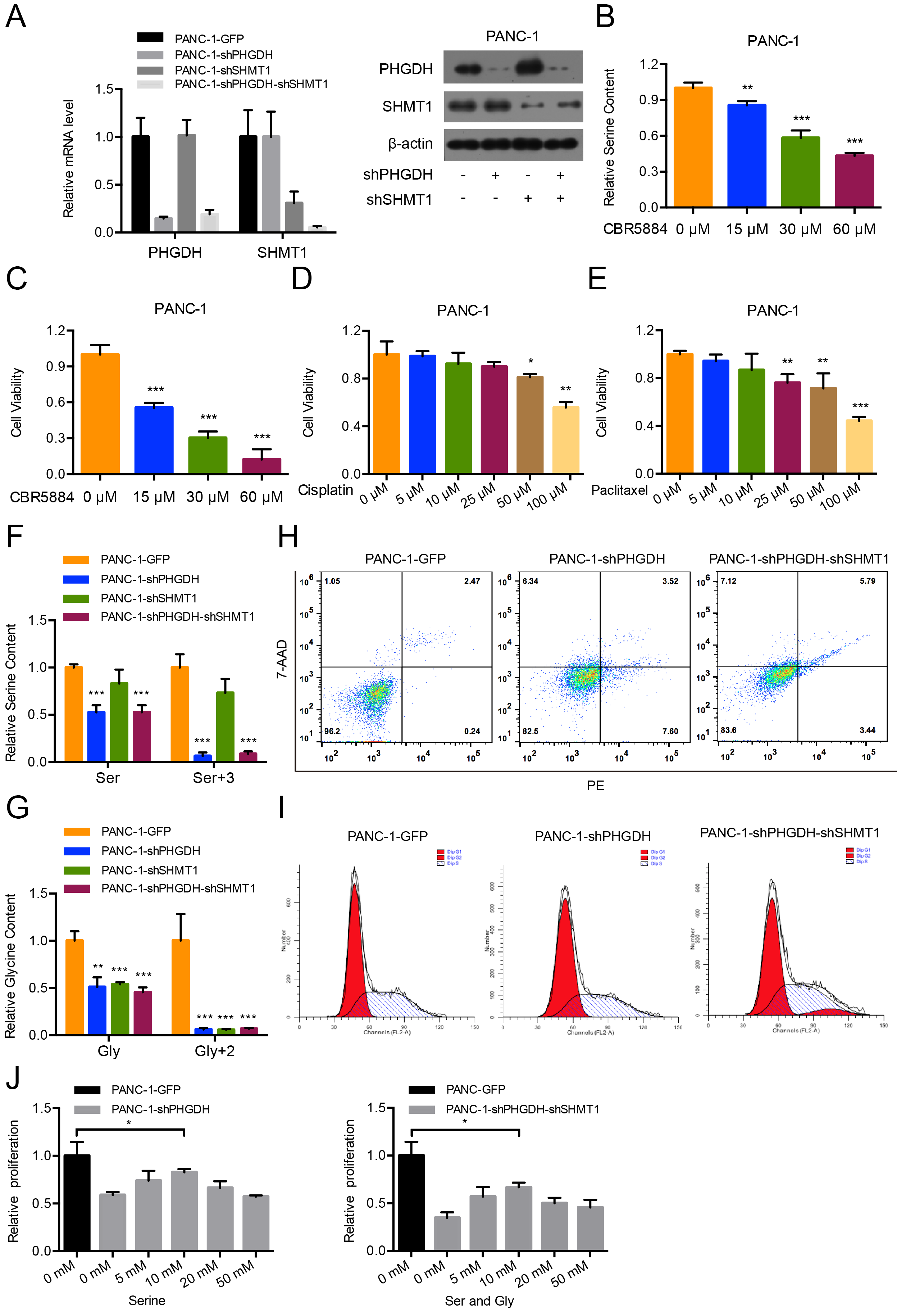
**

**Figure S2. Reduction of serine caused by PHGDH knockdown induces apoptosis and inhibits cell proliferation.**

(A) qRT-PCR and western blot confirmation of PHGDH and/or SHMT1 knockdown in PANC-1 cells. (B) Relative intracellular serine contents of PANC-1 cells under the treatment of PHGDH inhibitor CBR5884. (C) (D) and (E) Relative proliferation results of PANC-1 cells under the treatments of PHGDH inhibitor CBR5884, cisplatin and paclitaxel. (F) and (G) Relative total and labeled serine and glycine contents in the constructed PANC-1 cells. (H) and (I) Apoptosis and cell cycle analysis of the constructed PANC-1 cells. (J) Relative proliferation results of the constructed PANC-1 cells. Each cell line was respectively pre-cultured in serine, glycine or serine and glycine free culture media for 12 hours and then respectively added 0, 5, 10, 20, 50 mM serine or serine and glycine into the indicated cell cultures. Data are representative of at least three independent experiments. p value: Student’s t-test; *p < 0.05, **p < 0.01, ***p < 0.001. Columns: mean; bars: SD.

**Figure S3.**


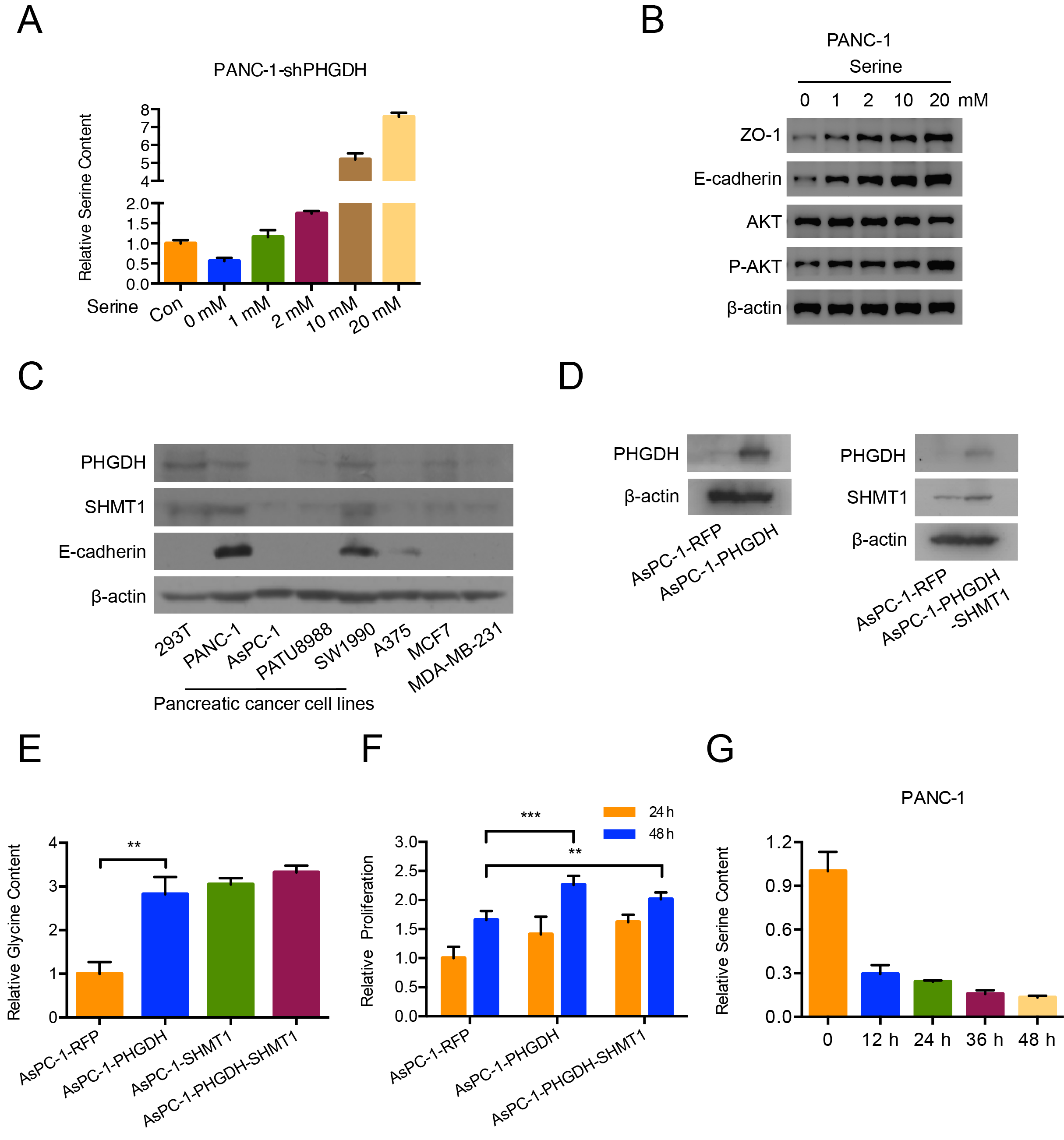


**Figure S3. Serine supply promotes the indicated proteins expression; PHGDH overexpression increases the intracellular glycine contents and promotes the proliferation of AsPC-1 cells.**

(A) Relative intracellular serine contents of PANC-1-shPHGDH cells under the normal and serine supply conditions. PANC-1-shPHGDH cells were pre-cultured in serine free culture media and then respectively added 0, 1, 2, 10, 20 mM serine into the media. (B) Serine supply promoted the indicated proteins expression in PANC-1 cells. (C) Western blot analysis to identify the PHGDH low-expressing cell line. (D) Western blot confirmation of PHGDH and PHGDH-SHMT1 overexpression in AsPC-1 cells. (E) Relative glycine contents in the constructed AsPC-1 cells. (F) Relative proliferation results of the constructed AsPC-1 cells for 24 and 48-hour cultures. (G) Relative intracellular serine contents of PANC-1 cells under the serine starvation condition for 48 hours. Data are representative of at least three independent experiments. p value: Student’s t-test; **p < 0.01, ***p < 0.001. Columns: mean; bars: SD.

**Figure S4.**

**
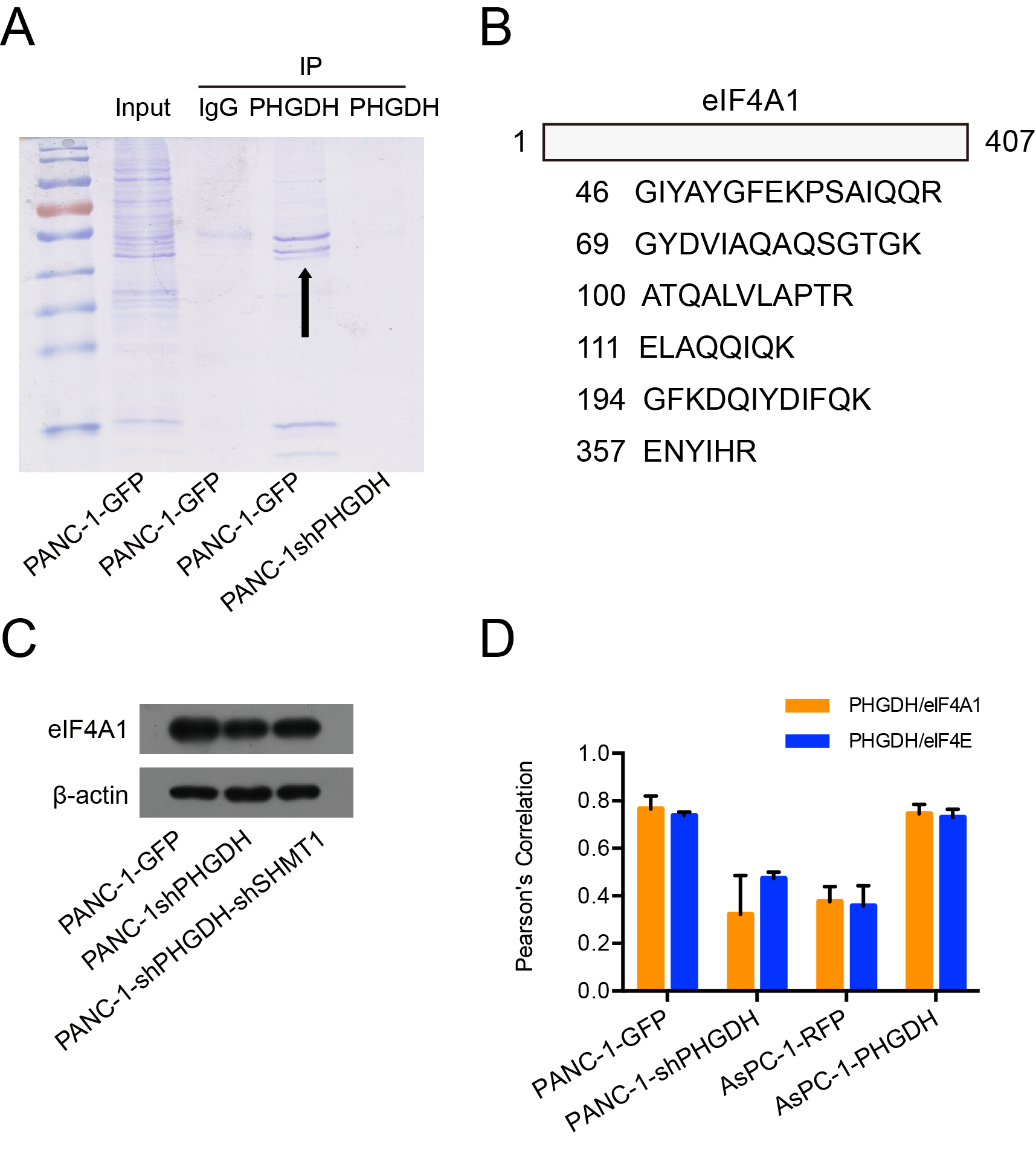
**

**Figure S4. PHGDH interacts with eIF4A1 and eIF4E.**

(A) Immunopercipitation results for PHGDH interacting proteins detection. The interacted proteins were separated by SDS-PAGE. (B) Six peptides spanning the sequence of eIF4A1 were identified by mass spectrometry. (C) Western blot analysis of the eIF4A1 expression levels in the constructed PANC-1 cells. (D) Quantified co-localization coefficient results of the Figures 4D and E. Data are representative of at least three independent experiments.

**Figure S5.**

**
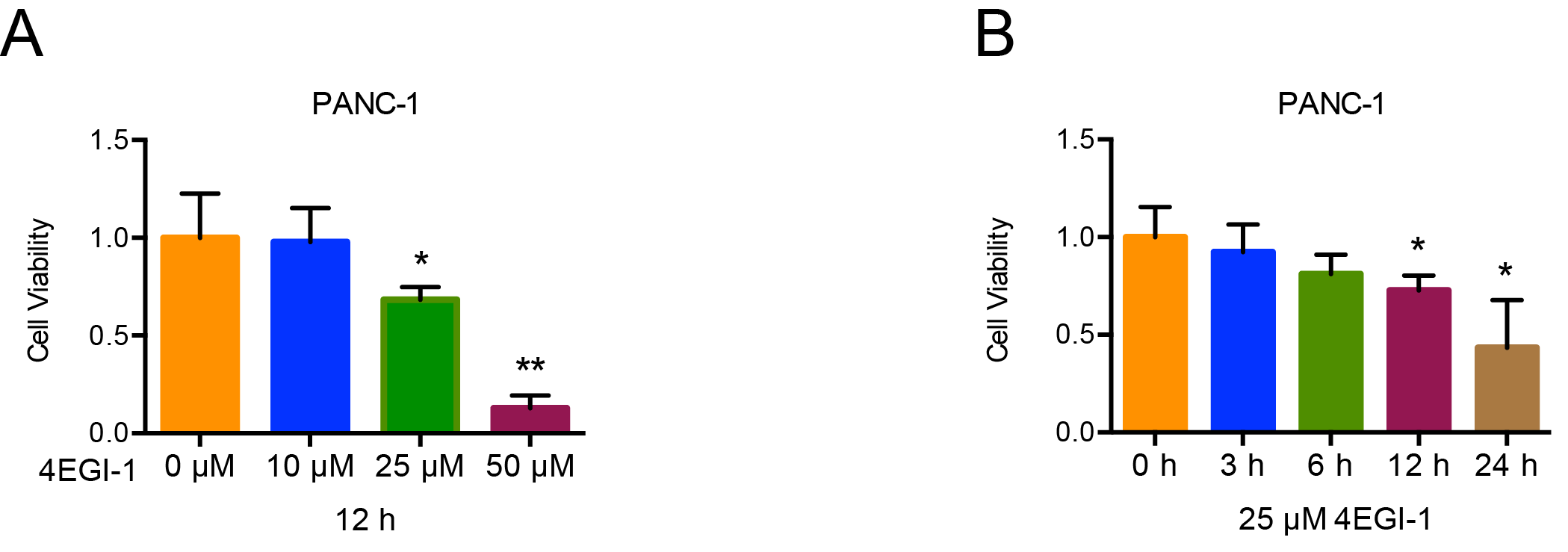
**

**Figure S5. Cytotoxicity test of 4EGI-1 in PANC-1 cells**

(A) Relative cell viability of PANC-1 cells under the treatments of 0, 10, 25, 50 μM 4EGI-1 for 12 hours. (B) Relative cell viability of PANC-1 cells under the treatments of 25 μM 4EGI-1 for 0, 3, 6, 12 and 24 hours. Data are representative of at least three independent experiments. p value: Student’s t-test; *p < 0.05, **p < 0.01.Columns: mean; bars: SD.

**Figure S6.**

**
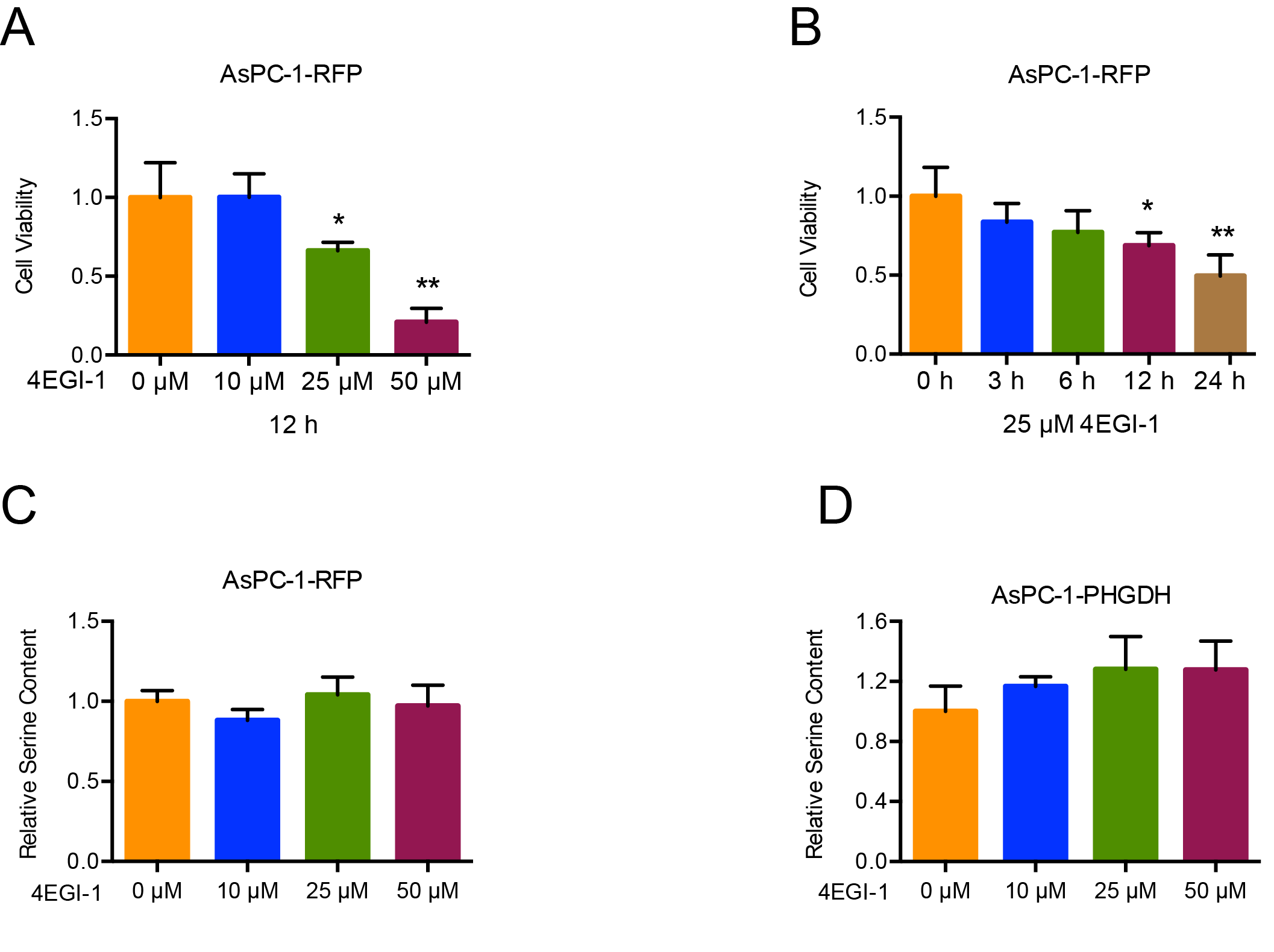
**

**Figure S6. Cytotoxicity test of 4EGI-1 and its effect on serine contents in the constructed AsPC-1 cells.**

(A) Relative cell viability of AsPC-1-RFP cells under the treatments of 0, 10, 25, 50 μM 4EGI-1 for 12 hours. (B) Relative cell viability of AsPC-1-RFP cells under the treatments of 25 μM 4EGI-1 for 0, 3, 6, 12 and 24 hours. (C) and (D) Relative intracellular serine contents in AsPC-1-RFP and AsPC-1-PHGDH cells under the treatments of 0, 10, 25, 50 μM 4EGI-1. Data are representative of at least three independent experiments. p value: Student’s t-test; *p < 0.05, **p < 0.01.Columns: mean; bars: SD.

**Figure S7.**


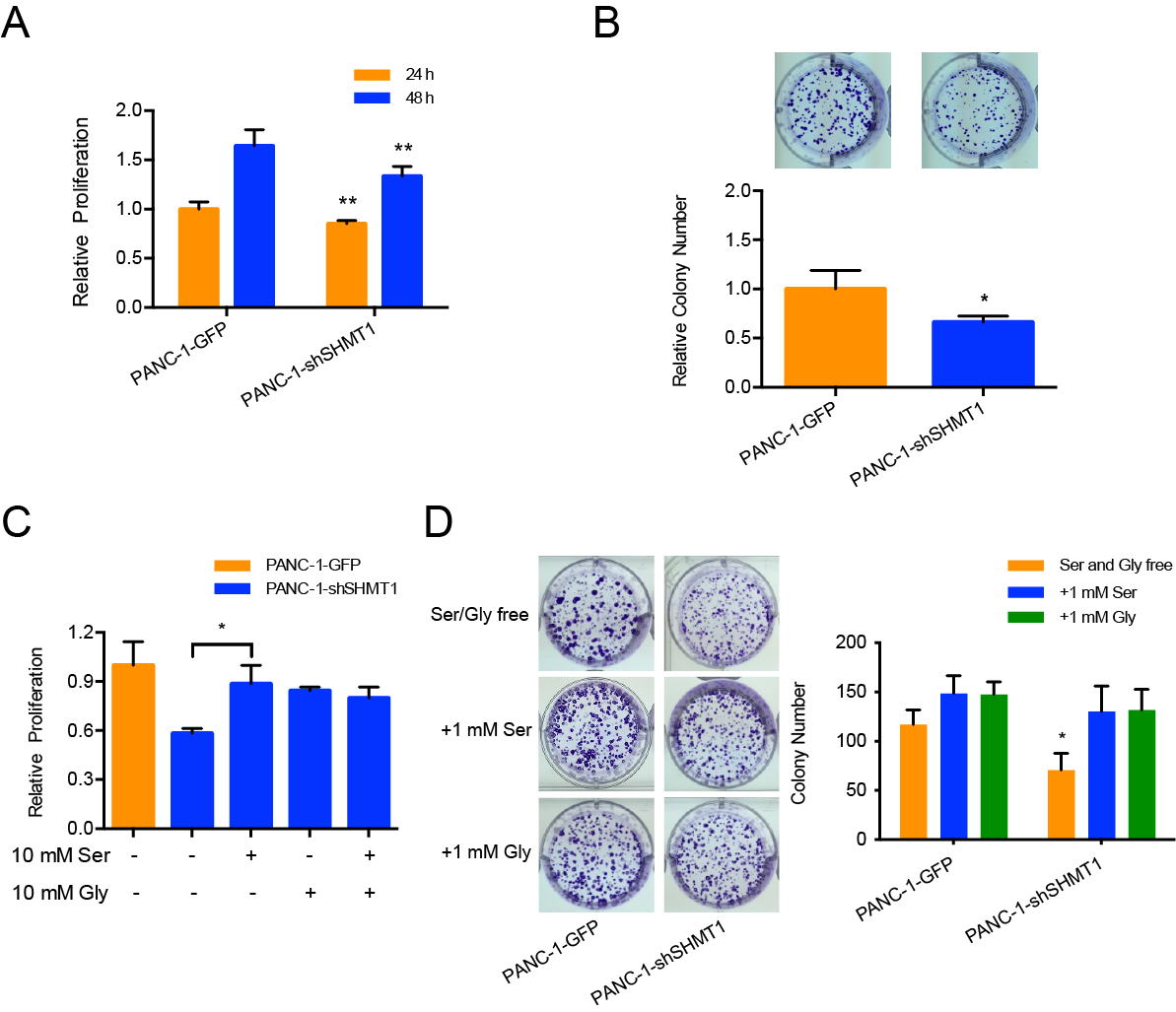


**Figure S7. Serine or glycine supplement completely rescues the impaired cell proliferation and colony formation caused by SHMT1 knockdown.**

(A) Relative proliferation results of PANC-1-GFP and PANC-1-shSHMT1 cells for 24 and 48-hour cultures. (B) Colony formation results of PANC-1-GFP and PANC-1-shSHMT1 cells under the normal conditions. (C) Relative proliferation results of PANC-1-GFP and PANC-1-shSHMT1 cells under the treatments of 10 mM serine and/or 10 mM glycine. (D) Colony formation results of PANC-1-GFP and PANC-1-shSHMT1 cells under the conditions of serine and glycine free (up), 1 mM serine supply (middle) and 1 mM glycine supply (down) respectively. Data are representative of at least three independent experiments. p value: Student’s t-test; *p < 0.05. Columns: mean; bars: SD.
